# Nitazoxanide in Patients Hospitalized With COVID-19 Pneumonia: A Multicentre, Randomized, Double-Blind, Placebo-Controlled Trial

**DOI:** 10.3389/fmed.2022.844728

**Published:** 2022-04-13

**Authors:** Patricia R. M. Rocco, Pedro L. Silva, Fernanda F. Cruz, Paulo F. G. M. M. Tierno, Eucir Rabello, Jéfiton Cordeiro Junior, Firmino Haag, Renata E. de Ávila, Joana D. G. da Silva, Mariana M. S. Mamede, Konrad S. Buchele, Luiz C. V. Barbosa, Anna C. Cabral, Antônio A. F. Junqueira, João A. Araújo-Filho, Lucianna A. T. J. da Costa, Pedro P. M. Alvarenga, Alexandre S. Moura, Ricardo Carajeleascow, Mirella C. de Oliveira, Roberta G. F. Silva, Cynthia R. P. Soares, Ana Paula S. M. Fernandes, Flavio Guimarães Fonseca, Vidyleison Neves Camargos, Julia de Souza Reis, Kleber G. Franchini, Ronir R. Luiz, Sirlei Morais, Carlos Sverdloff, Camila Marinelli Martins, Nathane S. Felix, Paula Mattos-Silva, Caroline M. B. Nogueira, Dayene A. F. Caldeira, Paolo Pelosi, José R. Lapa-e-Silva

**Affiliations:** ^1^Carlos Chagas Filho Institute of Biophysics, Federal University of Rio de Janeiro, Rio de Janeiro, Brazil; ^2^Hospital Municipal de Barueri Dr. Francisco Moran, Barueri, Brazil; ^3^Hospital da Força Aérea do Galeão, Rio de Janeiro, Brazil; ^4^Hospital Regional de Sorocaba Dr. Adib D Jatene- Bata Branca, São Paulo, Brazil; ^5^Hospital Geral de São Mateus – Dr. Manoel Bifulco, São Mateus, Brazil; ^6^Hospital Eduardo Menezes, Belo Horizonte, Brazil; ^7^Hospital Regional da Asa Norte, Brasília, Brazil; ^8^Hospital das Forças Armadas, Brasília, Brazil; ^9^Hospital Naval Marcilio Dias, Rio de Janeiro, Brazil; ^10^Hospital das Clínicas Luzia de Pinho Melo, Mogi das Cruzes, Brazil; ^11^Hospital Universitário Pedro Ernesto, Rio de Janeiro, Brazil; ^12^Hospital Central da Aeronáutica, Rio de Janeiro, Brazil; ^13^Hospital Estadual de Doenças Tropicais Dr. Anuar Auad, Anápolis, Brazil; ^14^Hospital Geral de Fortaleza, Fortaleza, Brazil; ^15^Hospital Mater Dei, Belo Horizonte, Brazil; ^16^Santa Casa de Misericórdia de Belo Horizonte, Belo Horizonte, Brazil; ^17^Complexo Hospitalar Municipal de São Caetano do Sul, Sao Caetano do Sul, Brazil; ^18^Complexo do Trabalhador de Curitiba, Curitiba, Brazil; ^19^Hospital da Força Aérea de São Paulo, São Paulo, Brazil; ^20^Hospital das Clínicas da Universidade Federal do Pernambuco, Recife, Brazil; ^21^Centro de Tecnologia de Vacinas, Universidade Federal de Minas Gerais, Belo Horizonte, Brazil; ^22^Brazilian Centre for Research in Energy and Materials, Campinas, Brazil; ^23^ATCGEN, Campinas, Brazil; ^24^AAC&T Research Consulting LTDA, Curitiba, Brazil; ^25^Department of Surgical Sciences and Integrated Diagnostics, University of Genoa, Genoa, Italy; ^26^Anesthesia and Critical Care, San Martino Policlinico Hospital, Istituto di Ricovero e Cura a Carattere Scientifico (IRCCS) for Oncology and Neurosciences, Genoa, Italy

**Keywords:** D-dimer, oxygenation, pneumonia, SARS-CoV-2, COVID-19

## Abstract

**Background:**

Nitazoxanide exerts antiviral activity *in vitro* and *in vivo* and anti-inflammatory effects, but its impact on patients hospitalized with COVID-19 pneumonia is uncertain.

**Methods:**

A multicentre, randomized, double-blind, placebo-controlled trial was conducted in 19 hospitals in Brazil. Hospitalized adult patients requiring supplemental oxygen, with COVID-19 symptoms and a chest computed tomography scan suggestive of viral pneumonia or positive RT-PCR test for COVID-19 were enrolled. Patients were randomized 1:1 to receive nitazoxanide (500 mg) or placebo, 3 times daily, for 5 days, and were followed for 14 days. The primary outcome was intensive care unit admission due to the need for invasive mechanical ventilation. Secondary outcomes included clinical improvement, hospital discharge, oxygen requirements, death, and adverse events within 14 days.

**Results:**

Of the 498 patients, 405 (202 in the nitazoxanide group and 203 in the placebo group) were included in the analyses. Admission to the intensive care unit did not differ between the groups (hazard ratio [95% confidence interval], 0.68 [0.38–1.20], *p* = 0.179); death rates also did not differ. Nitazoxanide improved the clinical outcome (2.75 [2.21–3.43], *p* < 0.0001), time to hospital discharge (1.37 [1.11–1.71], *p* = 0.005), and reduced oxygen requirements (0.77 [0.64–0.94], *p* = 0.011). C-reactive protein, D-dimer, and ferritin levels were lower in the nitazoxanide group than the placebo group on day 7. No serious adverse events were observed.

**Conclusions:**

Nitazoxanide, compared with placebo, did not prevent admission to the intensive care unit for patients hospitalized with COVID-19 pneumonia.

**Clinical Trial Registration:**

Brazilian Registry of Clinical Trials (REBEC) RBR88bs9x; ClinicalTrials.gov, NCT04561219.

## Introduction

COVID-19 is a multiple organ disease (1); patients presenting with moderate to severe symptoms may require intensive care unit (ICU) admission (2, 3). To date, pharmacological therapies with antiviral properties have presented modest results (4–6). Nitazoxanide has shown antiviral and anti-inflammatory effects (7–10). In mild COVID-19, nitazoxanide significantly reduced the viral load with no serious adverse events (7). More recently, a randomized, double-blind placebo-controlled clinical trial provides evidence that treatment of outpatients with mild or moderate COVID-19 with nitazoxanide may reduce the progression to severe illness, thus suggesting larger trials with adequate statistical power to confirm this hypothesis (11). Nitazoxanide and its metabolite (tizoxanide) decreased inflammation *in vitro* and *in vivo* (8, 9). Nitazoxanide alone reduced the duration of symptoms in patients with acute uncomplicated influenza (12). In a proof-of-concept pilot trial, nitazoxanide decreased the mean time to hospital discharge, viral load, and inflammatory mediators in patients with COVID-19 (10). Moreover, nitazoxanide is available at low cost particularly in low- and middle-income countries. We hypothesized that nitazoxanide could effectively reduce the number of patients with COVID-19 pneumonia admitted to the ICU due to the need for invasive mechanical ventilation. Secondary outcomes included clinical improvement, time to hospital discharge, oxygen requirements, death, radiological and laboratory findings, and adverse events during hospitalization until day 14.

## Methods

### Study Design

A double-blind, placebo-controlled trial was conducted in 19 hospitals in Brazil (Supplementary Material). This study adheres to the Declaration of Helsinki and the Brazilian National Commission for Research Ethics (CAAE:30662420.0.1001.0008) and individual Ethics Committees of all participating sites approved this study. This trial was registered in the Brazilian Registry of Clinical Trials (REBEC) number RBR-88bs9x and ClinicalTrials.gov number NCT04561219. This report follows the Consolidated Standards of Reporting Trials (CONSORT) guideline (13). The final protocol, amendments and changes to the trial protocol are detailed in Supplementary Material.

### Patients

Consecutive adult patients (≥18 years), requiring supplemental oxygen (peripheral oxygen saturation [SpO_2_] < 93%) and admitted to hospital with COVID-19 symptoms associated with a chest computed tomography (CT) findings suggestive of viral pneumonia or a positive nasopharyngeal swab test for SARS-CoV2 (RT-PCR) were eligible for inclusion. The inclusion and exclusion criteria are listed in the Supplementary Material.

### Randomization and Masking

With the aid of a computer-generated random number list, patients were randomly assigned (1:1) to receive placebo or nitazoxanide [500 mg oral solution, 20 mg/ml (25 ml), three times daily for 5 days], dispensed by the pharmacist at each hospital. Patients, treating clinicians, trial personnel, and outcome assessors were blinded to group assignment. Placebo and nitazoxanide were color-matched to ensure that assessors were unaware of the group allocation at all times (see the Supplementary Material for additional details regarding the trial design).

### Procedures

On day 1 (baseline), patients were assessed for eligibility. Written informed consent was obtained from all patients or from a legal representative (Supplementary Material). A nasopharyngeal swab was collected for RT-PCR testing. Site investigators performed a comprehensive physical examination, recording levels and type of oxygen supplementation and concomitant medications. Blood samples were taken (Supplementary Material). Adverse events, regardless of severity, were monitored throughout the trial by reviewing the electronic medical records, physical examination findings, vital signs, and laboratory tests from enrolment until day 14. Study data were entered directly into electronic case-report forms (REDCap) (14) and clinical trial management system (ATCGen) by the site investigator and validated by monitoring staff from ATCGen.

### Primary Outcome

The primary outcome was ICU admission due to the need for invasive mechanical ventilation at any point during hospitalization until day 14.

### Secondary Outcomes

The key secondary outcome measure was clinical status assessed daily according to an 8-point ordinal scale until day 14 (15) using the following categories: (1) not hospitalized, no limitation of activities; (2) not hospitalized, limitation of activities; (3) hospitalized, not requiring supplemental oxygen or ongoing medical care (used if hospitalization was extended for infection-control reasons); (4) hospitalized, not receiving supplemental oxygen, but requiring ongoing medical care (COVID-19–related or other medical conditions); (6) hospitalized, receiving supplemental oxygen; (6) hospitalized, receiving high-flow oxygen through a nasal cannula; (7) hospitalized, receiving invasive mechanical ventilation or extracorporeal membrane oxygenation (ECMO); (8) death. Time to recovery was defined as the period from beginning of therapy to discharge from the hospital over a 14-day follow-up.

Additional secondary outcomes included (1) the proportion of patients with clinical improvement [time to improve two categories on the aforementioned 8-point ordinal scale of clinical status at baseline (day 1), and on days 3, 5, 7, and 14]; (2) the number of patients discharged (ordinal scale 1–2); (3) oxygen requirement (ordinal scale 5–6); (4) the number of deaths (ordinal scale 8) from randomization until day 14; (5) duration of symptoms suggestive of COVID-19, including dry cough, productive cough, sore throat, and shortness of breath; (6) number of patients with negative RT-PCR test on day 7 and percentage reduction in viral RNA load on nasopharyngeal swab specimens (from day 1 until day 7); (7) clinical (body temperature, respiratory rate, and SpO_2_) and laboratory data (hemoglobin levels, WBC count, neutrophils, lymphocytes, platelets, C-reactive protein, D-dimer, ferritin, lactate dehydrogenase); (8) chest CT score (0–25%, 26–50%, 51–75%, and 76–100% of lung tissue compromised).

### Statistical Analyses

The statistical analysis plan was completed before the end of the study and unblinding of the study results and is available in the Supplementary Material.

Around 15% of patients hospitalized with COVID-19 were admitted to the ICU in March 2020. If 5% of hospitalized patients treated with nitazoxanide would be admitted to the ICU, with an alpha error of 5%, a statistical power of 90%, and two-tailed test, 198 participants per group would be required. Estimating a dropout rate of about 20%, our target sample size was set at 247 participants/group, which made up the intention-to-treat [ITT] population.

Descriptive statistics were used for demographic, laboratory, and clinical data. Fisher's exact test was used for qualitative variables. The Mann-Whitney *U* test or Student's *t*-test was used for comparisons between groups.

To evaluate the response to nitazoxanide, we separated the patients into three different groups: (1) ITT: the intention-to-treat population, all eligible patients, (2) mITT: the modified intention-to-treat population (ITT population fully treated who completed the 14-day analysis), and (3) the mITT-positive population (patients who completed the 14-day analysis and had a positive nasopharyngeal swab RT-PCR test for SARS-CoV2). Patients who dropped out or died were included in the ITT population, but not in the mITT or mITT-positive population. Safety analyses were done in the ITT and mITT populations.

For the primary outcome, the odds ratio (OR) was derived from a mixed-effect ordinal logistic regression, assuming proportional ORs, adjusted for age, sex, body mass index (BMI), and time from symptom onset to randomization. The Kaplan-Meier method was used for time-to-event analyses and compared with a log-rank test. The hazard ratios (HRs) and 95% confidence intervals (CIs) for the cumulative incidence of ICU admission in both groups were estimated using a Cox proportional regression model. The proportional hazards assumption was tested graphically using a log-log plot; there was no evidence to reject the assumption. Similar analyses were done for the ITT, mITT, and mITT-positive populations.

For the secondary outcomes, Kaplan-Meier survival curves were constructed to show clinical improvement, hospital discharge, oxygen requirement, and cumulative mortality over the 14-day period. Adjusted ORs were calculated using the same method used for the primary outcome. There was a single primary hypothesis test. For secondary outcomes, no adjustments for multiplicity were made.

Adverse events were described as absolute and relative frequencies by the type of event. Chi-squared tests were used to evaluate adverse events in the mITT and ITT populations.

Using the eight-point ordinal scale, treatment effect was evaluated at each time point; the frequency of each category in each group was recorded day-by-day and compared with the chi-squared test.

Type of oxygen support, respiratory symptoms, use of concomitant medications, and viral load were analyzed by calculating the percentage difference between groups, with the chi-squared test used to compare treatments day-by-day and the McNemar or Wilcoxon tests to compare changes between days (see Supplementary Material).

The deletion method (pairwise deletion) was used rather than imputation to eliminate missing data; this is particularly advantageous for samples where there is a large volume of data and values can be deleted without significantly distorting the readings. Pairwise deletion is the process of eliminating information when a particular data point, vital for testing, is missing. Pairwise deletion saves more data than likewise deletion because the former only deletes entries where variables were necessary for testing, whereas the latter deletes entire entries if any data are missing, regardless of their importance. Sensitivity analyses were performed to compare variables for the ITT versus the mITT and mITT-positive populations to verify if the process of elimination changed the profile and results.

To evaluate the effects of treatment on ICU admission, forest plots were constructed calculating ORs and 95%CIs for specific subgroups of patients according to the following variables: age, sex, BMI, time from symptom onset until randomization, SpO_2_ and chest CT score at day 1, and corticosteroid use during hospitalization.

All statistical analyses were performed in R (16), and a two-tailed *p*-value < 0.05 was considered significant.

## Results

**Figure 1:**
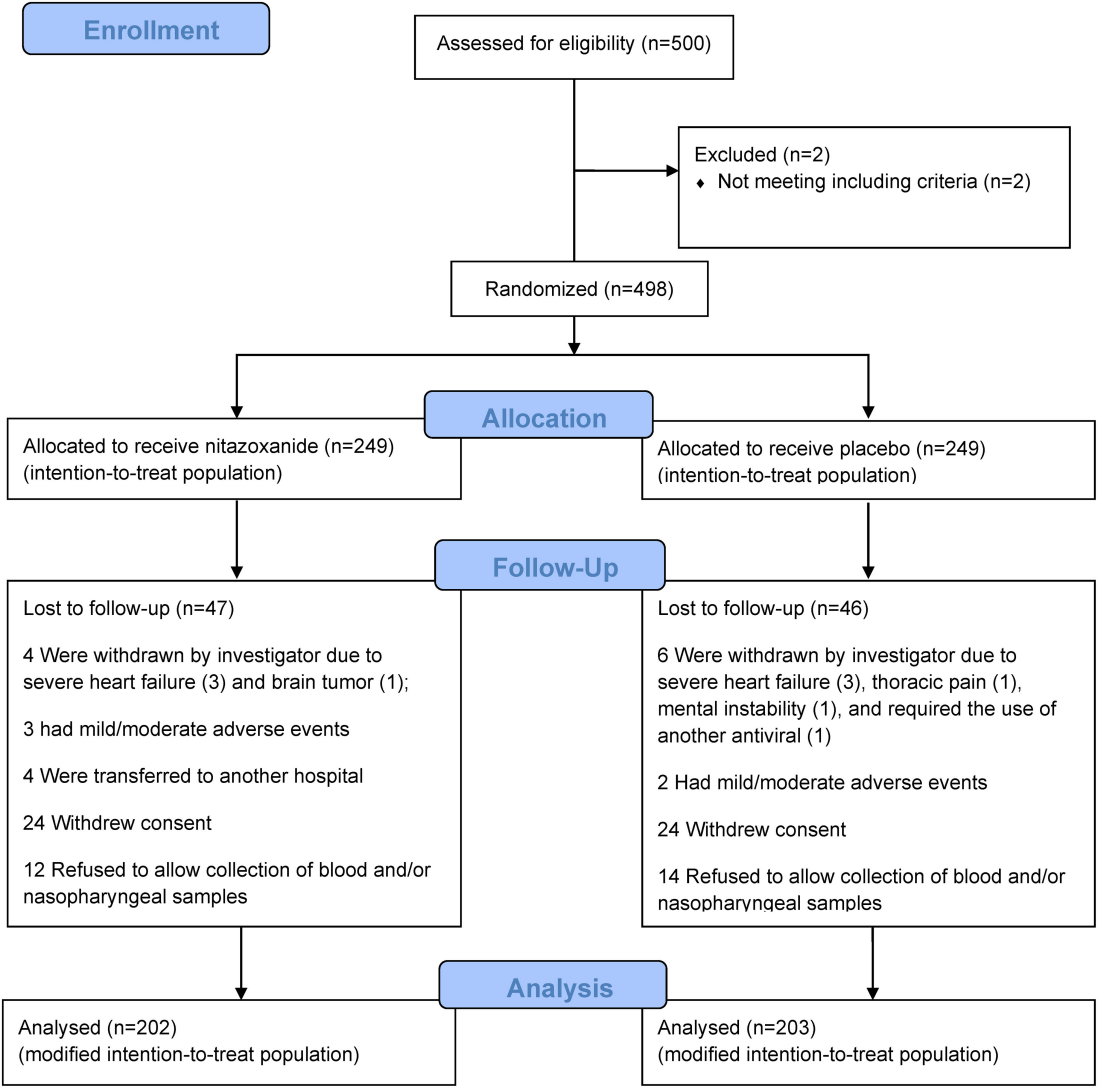
Enrolment, randomization, follow-up, and treatment. Five hundred patients were assessed for eligibility at the study sites. Of these, 498 underwent randomization, 2 patients were excluded for not meeting the inclusion criteria. After randomization (*n* = 249/group), patients were excluded because they were withdrawn by the investigator, had mild/moderate adverse events, were transferred to another hospital, withdrew consent at any time during the study, refused to allow collection of blood and/or nasopharyngeal samples. Therefore, the modified intention-to-treat population consisted of 202 patients in the nitazoxanide group and 203 patients in the placebo group.

From April 20 to October 2, 2020, 500 patients were assessed for eligibility at the study sites. Two patients did not meet the inclusion criteria; 498 underwent randomization (249 to the nitazoxanide group and 249 to the placebo group), representing the ITT population (Figure 1). Of the 249 patients, 47 patients in the nitazoxanide group and 46 patients in the placebo group were lost to follow-up. Therefore, the mITT population consisted of 202 patients in the nitazoxanide group and 203 patients in the placebo group. Among these 405 patients, 367 had a positive SARS-CoV-2 RT-PCR test and constituted the mITT-positive population (183 in the nitazoxanide group and 184 in the placebo group).

### Baseline (Day 1) Characteristics

In the mITT group, the median age was 56 years [interquartile range (IQR), 46–67 years], 61% were men, 45% represented mixed ethnicity, and 35% were obese (Table 1). The median (IQR) number of days from symptom onset to the first dose of nitazoxanide or placebo was 7 (IQR, 5–10 days). The most common comorbidity was hypertension (36%). Patients were on angiotensin-II receptor antagonists (16%) or angiotensin-converting enzyme inhibitors (20%). At day 1, 91% had positive nasopharyngeal swabs. Dry cough and shortness of breath were the most common symptoms and fever (>38°C) was present in only 7% of patients at hospital admission. All patients received nitazoxanide or placebo as appropriate on the day of randomization. The three populations were well matched according to day 1 characteristics (Table 1; Supplementary Material).

**Table 1:** Demographic and disease characteristics of the patients at baseline in the mITT population.

**Characteristics**	**All patients (*n* = 405)**	**Nitazoxanide (*n* = 202)**	**Placebo (*n* = 203)**
Age (years), median (IQR)	56 (46–67)	56 (46–69)	56 (45–66)
**Age range**, ***n*** **(%)**			
18-40 years	58 (14)	29 (14)	29 (14)
41-59 years	187 (46)	92 (46)	95 (47)
≥60 years	160 (40)	81 (40)	79 (39)
Male sex, *n* (%)	248 (61)	117 (58)	131 (65)
**Ethnicity**, ***n*** **(%)**			
Mixed	183 (45)	90 (45)	93 (46)
White	175 (43)	87 (43)	88 (43)
Black	34 (8)	17 (8)	17 (8)
Asian	10 (3)	7 (4)	3 (2)
Other	3 (1)	1 (0.5)	2 (1)
**BMI**, ***n*** **(%)**
<29.9 kg/m^2^	264 (65)	133 (66)	131 (65)
≥30.0 kg/m^2^	141 (35)	69 (34)	72 (36)
Time from symptom onset to randomization (days), median (IQR)	7 (5–10)	7 (5–10)	7 (5–10)
**Coexisting condition**, ***n*** **(%)**			
Hypertension	146 (36)	73 (36)	73 (36)
Diabetes mellitus	90 (22)	42 (21)	48 (24)
Asthma	8 (2)	5 (3)	3 (2)
Chronic obstructive pulmonary disease	5 (1)	3 (2)	2 (1)
Human immunodeficiency virus infection	3 (1)	2 (1)	1 (0.5)
None	226 (56)	114 (56)	112 (55)
**Concomitant medications**, ***n*** **(%)**			
Angiotensin-II receptor antagonists	63 (16)	36 (18)	27 (13)
Angiotensin-converting enzyme inhibitors	79 (20)	41 (20)	38 (19)
**Symptom at diagnosis**, ***n*** **(%)**			
Dry cough	332 (82)	163 (81)	169 (83)
Productive cough	34 (8)	21 (10)	13 (6)
Sore throat	6 (2)	3 (2)	3 (2)
Shortness of breath	304 (75)	162 (80)	142 (70)
**Clinical parameters at hospital admission**			
Temperature (°C), median (IQR)	36 (36–37)	36 (36–37)	36 (36–37)
Respiratory rate (breaths/min), median (IQR)	21 (19–24)	21 (19–24)	21 (19–24)
SpO_2_ (%), median (IQR)	92 (90–93)	92 (89–93)	92 (90–93)
**Diagnosis of SARS-CoV-2 infection**, ***n*** **(%)**			
Nasopharyngeal swab, positive	367 (91)	183 (91)	184 (91)
**Laboratory parameters at hospital admission**			
Hb (g/dl), median (IQR)	13.5 (12.1-14.6)	13.5 (11.8-14.5)	13.4 (12.1-14.6)
WBC (× 10^3^/ml), median (IQR)	7.7 (5.9-9.8)	7.9 (6.3-9.8)	7.5 (5.8-9.8)
Neutrophils (× 10^3^/ml), median (IQR)	5.8 (4.2–7.5)	6.0 (4.3–7.6)	5.5 (4.1–7.2)
Lymphocytes (× 10^3^/ml), median (IQR)	1.9 (1.4–2.5)	1.9 (1.4–2.5)	1.9 (1.4–2.6)
Platelets (× 10^3^/ml), median (IQR)	215 (166–284)	218 (169–286)	216 (165–290)
C-reactive protein (mg/L), median (IQR)	120 (76–149)	116 (74–146)	121 (85–149)
D-dimer (mg/L), median (IQR)	1,135 (564–1,737)	1,152 (592–1,686)	1,121 (545–1,956)
Ferritin (mg/L), median (IQR)	509 (282–747)	485 (240–747)	480 (273–727)
Lactate dehydrogenase (IU/L), median (IQR)	296 (184–425)	300 (184–433)	270 (180–397)
**Chest CT score**, ***n*** **(%)**			
0–25%	0 (0)	0 (0)	0 (0)
26–50%	248 (61)	121 (60)	127 (63)
51–75%	94 (23)	47 (23)	47 (23)

*Coexisting conditions were coded according to the terms used in the Medical Dictionary for Regulatory Activities (MedDRA), version 23.0. Descriptive statistics with simple and relative frequencies for qualitative variables and median and interquartile range for continuous variables. Baseline, immediately before the administration of nitazoxanide or placebo on day 1. Ethnicity was self-reported by the patients*.

*BMI, body mass index (weight in kilograms divided by the square of the height in metres); COVID-19, coronavirus disease 2019; CT, computed tomography; Hb, hemoglobin; IQR, interquartile range; mITT, modified intention-to-treat; IU, international unit; SARS-CoV-2, severe acute respiratory syndrome coronavirus 2; WBC, white blood cell count*.

### Primary Outcome

**Figure 2:**
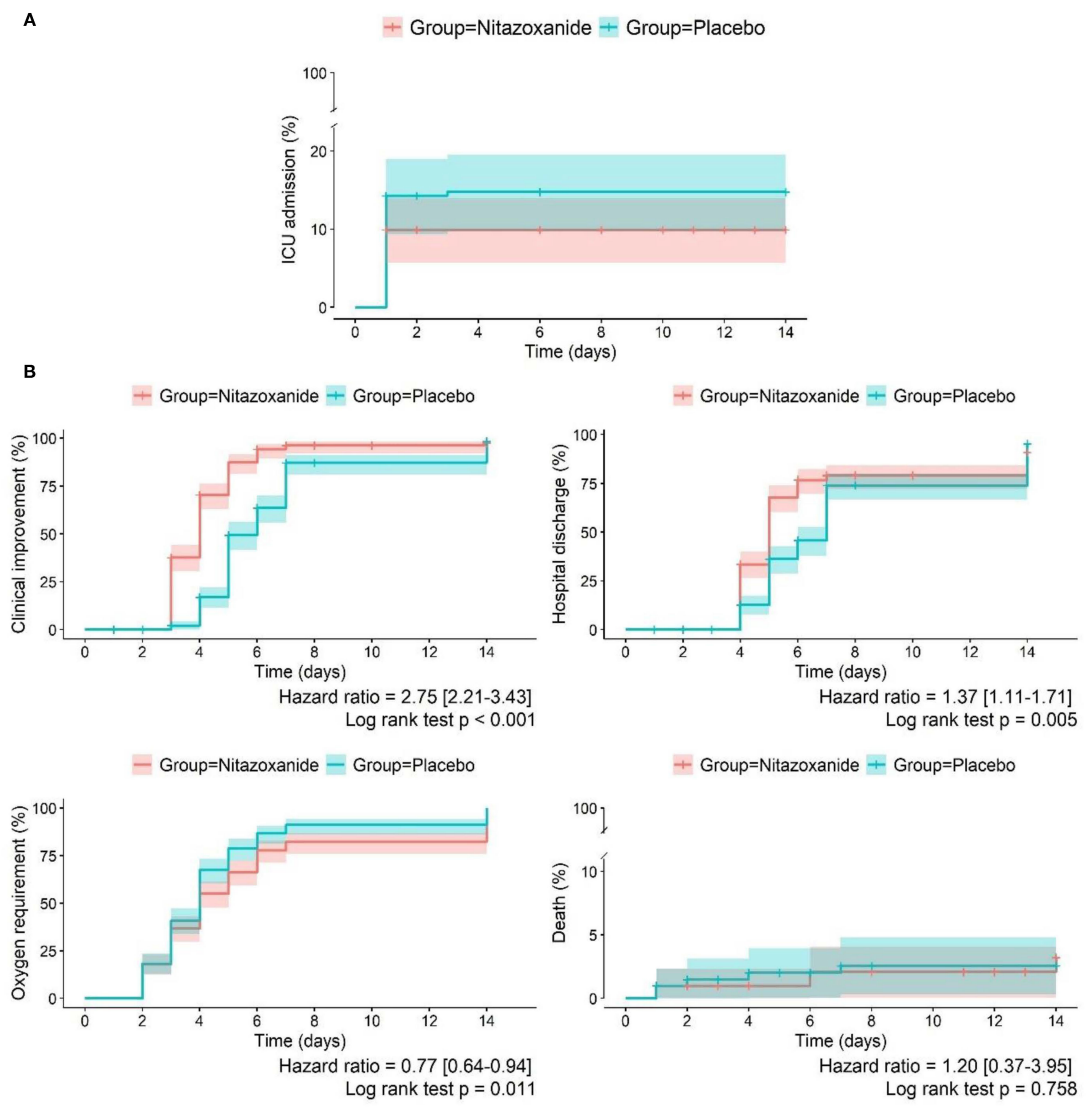
Primary and secondary outcomes in the nitazoxanide and placebo groups at the different time points. **(A)** Primary outcome: intensive care unit admission. **(B)** Secondary outcomes: clinical improvement, hospital discharge, oxygen requirement and death in the mITT population treated with nitazoxanide or placebo until day 14. The Kaplan-Meier curves and hazard ratios with corresponding 95% confidence intervals were calculated from a Cox proportional hazards model. mITT, modified intention-to-treat.

**Figure 3:**
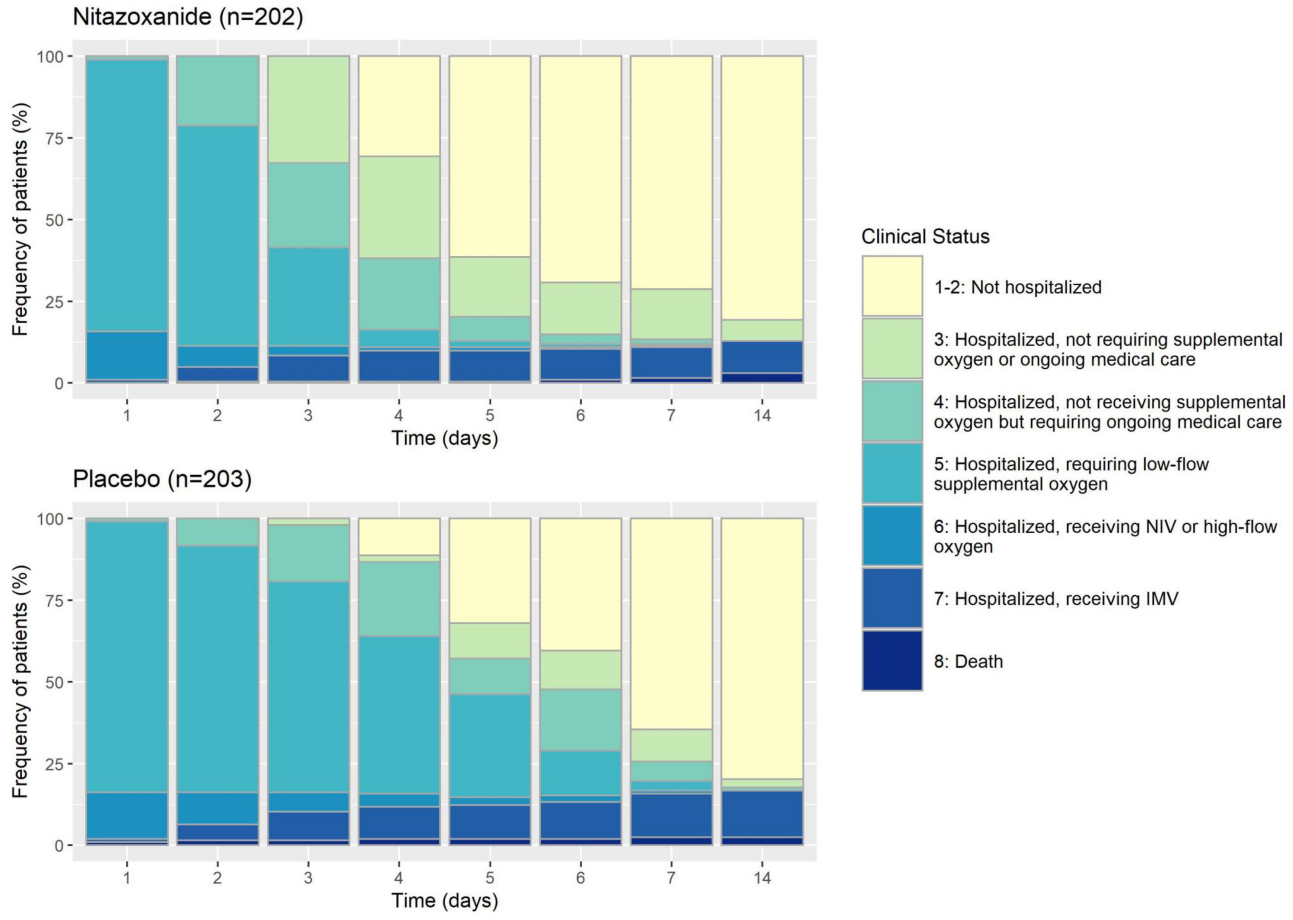
Eight-point ordinal scale of clinical status in the mITT (modified intention-to-treat) population treated with nitazoxanide or placebo until day 14. NIV, non-invasive ventilation; IMV, invasive mechanical ventilation.

In the mITT population, at day 14, 20 patients (10%) in the nitazoxanide group were admitted to the ICU requiring invasive mechanical ventilation, and 29 patients (15%) were admitted in the placebo group. The median (95% CI) Kaplan-Meier estimate of need for ICU admission was 13 (12, 13) days for the nitazoxanide group and 12 (11–13) days for the placebo group (HR [95% CI], 0.68 [0.38–1.20]; *p* = 0.179) (Table 2; Figures 2, 3; Supplementary Figure 1). The primary outcome was statistically similar across the ITT, mITT, and mITT-positive populations (Supplementary Material).

**Table 2:** Primary and secondary outcomes in the mITT population.

**Outcomes**	**Nitazoxanide (*n* = 202)**	**Placebo (*n* = 203)**	**Adjusted OR (95% CI)^**a**^**	***P*-value between groups**
**Primary outcome**				
**Intensive care unit admission due to the need for invasive mechanical ventilation, category 7 on the eight-point ordinal scale of clinical status**, ***n*** **(%)**				
Day 1	2 (1)	4 (2)	0.50 (0.09–2.87)	0.437
Day 3	16 (8)	18 (9)	0.84 (0.41–1.71)	0.630
Day 5	19 (10)	21 (11)	0.87 (0.45–1.97)	0.678
Day 7	19 (10)	27 (14)	0.66 (0.35–1.23)	0.192
Day 14	20 (10)	30 (15)	0.66 (0.36–1.21)	0.179
Kaplan-Meier estimate (days), mean (95% CI)	13 (12–13)	12 (11–13)		0.179
HR (95% CI)^b^	0.68 (0.38–1.20)			
**Secondary outcomes**				
**Clinical improvement (at least two categories on the eight-point ordinal scale of clinical status)**, ***n*** **(%)**				
Day 3	72 (36)	4 (2)	28.33 (10.07–79.70)	<0.001
Day 5	161 (80)	89 (43)	5.63 (3.56–8.92)	<0.001
Day 7	175 (87)	154 (76)	2.16 (1.28–3.66)	0.004
Day 14	176 (87)	167 (82)	1.52 (0.87–2.66)	0.141
Kaplan-Meier estimate (days), mean (95% CI)	4 (4–5)	7 (6–7)		<0.001
HR (95% CI)	2.75 (2.21–3.43)			
**Hospital discharge, category 1–2 on the 8-point ordinal scale of clinical status**, ***n*** **(%)**				
Day 1	0 (0)	0 (0)	–	–
Day 3	0 (0)	0 (0)	–	–
Day 5	124 (61)	65 (32)	3.60 (2.37–5.47)	<0.001
Day 7	144 (71)	131 (65)	1.50 (0.97–2.32)	0.066
Day 14	163 (81)	162 (80)	1.09 (0.66–1.81)	0.822
Kaplan-Meier estimate (days), mean (95% CI)	7 (6–7)	8 (7–8)		0.005
HR (95% CI)	1.37 (1.11–1.71)			
**Oxygen requirement**, ***n*** **(%)**				
Day 1	202 (100)	203 (100)	-	-
Day 3	135 (67)	196 (98)	0.04 (0.01–0.11)	<0.001
Day 5	40 (20)	112 (56)	0.17 (0.11–0.27)	<0.001
Day 7	24 (12)	48 (24)	0.42 (0.24–2.46)	0.002
Day 14	22 (11)	33 (17)	0.56 (0.31–1.03)	0.061
Kaplan-Meier estimate (days), mean (95% CI)	6 (5–6)	5 (4–5)		0.011
HR (95% CI)	0.77 (0.64–0.94)			
**Death, category 8 on the eight-point ordinal scale of clinical status**, ***n*** **(%)**				
Day 1	0 (0)	2 (1)	-	0.157
Day 3	1 (0.5)	3 (2)	0.25 (0.02–2.63)	0.251
Day 5	1 (0.5)	4 (2)	0.20 (0.02–1.90)	0.161
Day 7	3 (2)	5 (3)	0.48 (0.11–2.18)	0.341
Day 14	6 (3)	5 (3)	1.05 (0.29–3.74)	0.945
Kaplan-Meier estimate (days), mean (95% CI)	14 (13–14)	14 (13–14)		0.758
HR (95% CI)	1.20 (0.37–3.95)			

*Primary and secondary outcomes were analyzed in the modified intention-to-treat population. CI, confidence interval; HR, hazard ratio; OR, odds ratio*.

a *Adjusted OR calculated by multiple logistic regression assuming proportional ORs, adjusted for age range (<60 years, ≥60 years), sex, body mass index range (<30 kg/m^2^, ≥30 kg/m^2^), and time to symptom onset (≤7 days, >7 days)*.

b *Hazard ratio was calculated by means of a Cox proportional hazards model according to the moment of analysis*.

### Secondary Outcomes

Clinical improvement, defined as an increase of at least 2 points on the 8-ordinal scale for clinical status, was greater in the nitazoxanide group than in the placebo group from day 3 until day 7 (HR [95% CI], 2.75 [2.21–3.43], *p* < 0.0001). On day 14, no difference in clinical improvement was detected between the groups (Table 2; Figures 2, 3; Supplementary Material). The number of patients discharged from hospital (clinical status 1–2 on the 8-point ordinal scale) was also higher in the nitazoxanide group than in the placebo group from day 3 to day 7 (Table 2; Figures 2, 3; Supplementary Material). Patients who received nitazoxanide recovered a median of 1 day faster than patients treated with placebo (7 [6–7] days vs. 8 [7–8] days, *p* = 0.005; HR [95% CI], 1.37 [1.11–1.71]). On day 14, no significant differences were observed between the groups in the number of patients discharged from hospital (163 [80.7%] vs. 162 [79.8%]; *p* = 0.822) (Table 2; Figures 2, 3; Supplementary Material). From day 3 to day 7, nitazoxanide reduced oxygen requirement of any kind vs. placebo (HR [95% CI], 0.77 [0.64–0.94], *p* < 0.011) On day 14, no difference in oxygen requirement was observed between the groups. Overall, nitazoxanide reduced the time on supplemental oxygen by a median of 2 days compared to placebo (Table 2; Figures 2, 3; Supplementary Material). The number of deaths by day 14 did not differ between the nitazoxanide (6 [3.0%]) and placebo (5 [2.5%]) groups. The time to death did not differ between nitazoxanide and placebo (14 [13–14] days in both groups; *p* = 0.758; HR, 1.20 [0.37–3.95]) (Table 2; Figures 2, 3; Supplementary Material).

Nitazoxanide accelerated symptom resolution vs. placebo (Supplementary Material). The medications used during the study did not differ between the groups (Supplementary Material).

Of 405 patients, 367 (183 in the nitazoxanide group and 184 in the placebo group) were SARS-CoV-2 RT-PCR positive at enrolment. Viral load on nasopharyngeal swab reduced from day 1 to day 7 in both groups (Supplementary Material). On day 7, the viral load in the mITT-positive population did not reduce significantly in the nitazoxanide group vs. the placebo group (*p* = 0.083). All patients with negative viral load at baseline remained negative on day 7. Higher viral load at day 1 was associated with a trend toward greater reduction (*p* = 0.054) in the nitazoxanide group versus the placebo group (Supplementary Material)

SpO_2_ increased over time and differences were observed between the groups on day 3 (Supplementary Material). In the nitazoxanide group, C-reactive protein levels were lower on days 3 and 7 compared to placebo. On day 7, D-dimer, ferritin, and LDH levels were lower in the nitazoxanide vs. placebo group (Supplementary Material).

Lung involvement, as assessed by chest CT score, reduced to a greater extent after nitazoxanide vs. placebo over time (Supplementary Material).

Mild and moderate adverse events were experienced by patients in both groups during the 14-day course of therapy in the mITT and ITT populations (Table 3; Supplementary Material). The most common adverse events were diarrhea, headache, and nausea, with no significant differences between the groups.

**Table 3:** Adverse events in the mITT population.

	**Nitazoxanide (*n* = 202)**	**Placebo (*n* = 203)**	***P*-value^a^**
Number of participants with at least one adverse event, *n* (%)	70 (35)	70 (34)	0.971
Number of participants with two adverse events, *n* (%)	20 (10)	23 (11)	0.641
Number of participants with three or more adverse events, *n* (%)	27 (13)	21 (10)	0.347
**Specific adverse events**			
Diarrheal, *n* (%)	22 (11)	18 (9)	0.495
Headache, *n* (%)	33 (16)	45 (22)	0.137
Nausea, *n* (%)	19 (9)	13 (6)	0.263
Abdominal pain, *n* (%)	11 (5)	14 (7)	0.544
Abnormal color of urine, *n* (%)	8 (4)	6 (3)	0.580
Vomiting, *n* (%)	3 (1)	0 (0)	0.081
Pruritus, *n* (%)	2 (1)	1 (0)	0.559
Urticaria, *n* (%)	4 (2)	4 (2)	0.994

*All adverse events reported after nitazoxanide or placebo*.

a *p-value comparing proportions of adverse events between nitazoxanide and placebo*.

**Figure 4:**
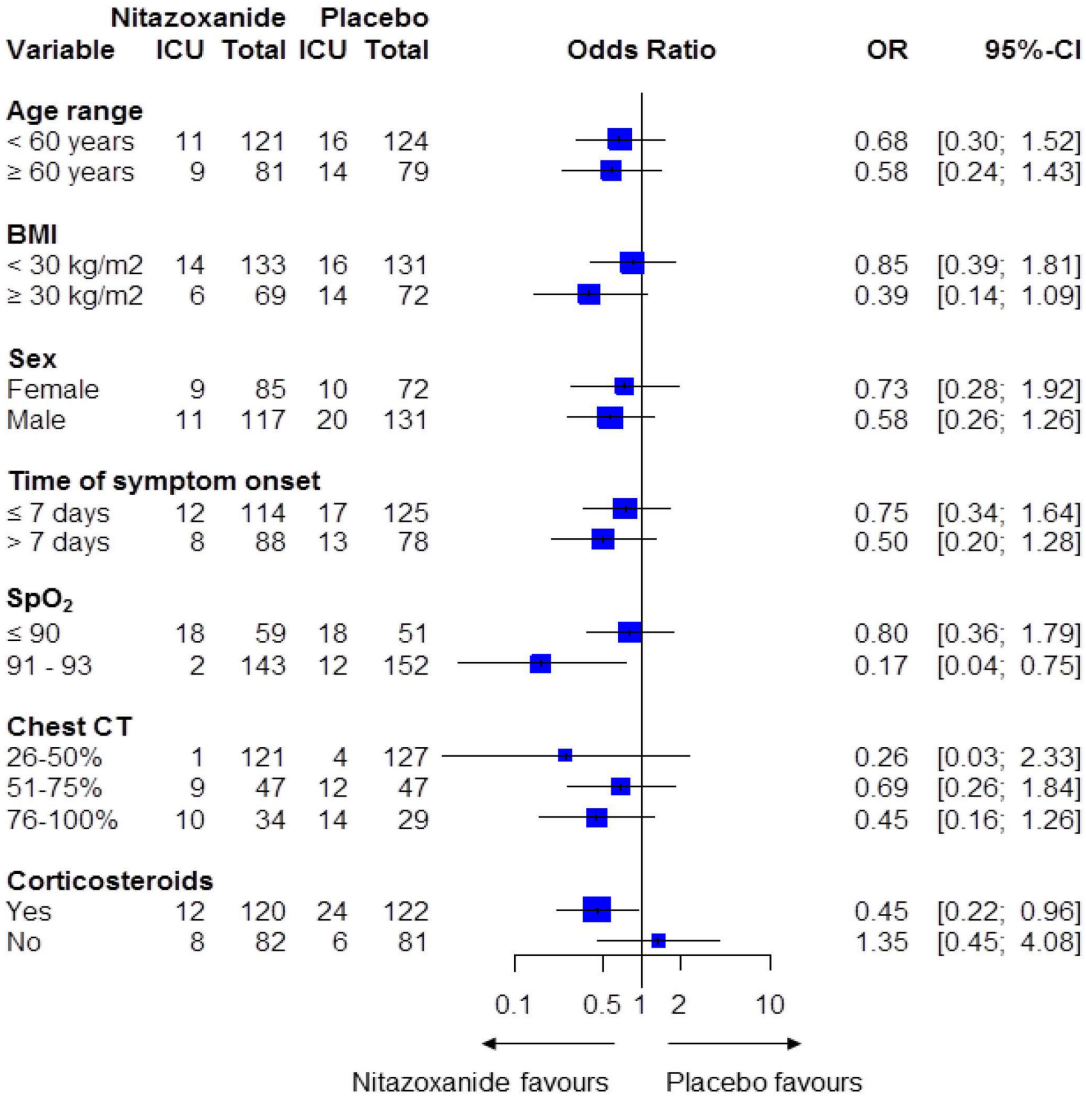
Forest plot according to the primary outcome in the nitazoxanide and placebo groups. BMI, body mass index; CI, confidence interval; OR, odds ratio; SpO_2_, peripheral saturation of oxygen. ORs and 95% CIs were calculated for each category individually.

Further analysis showed that hospitalized patients who presented with SpO_2_ higher than 90% at day 1 and were treated with nitazoxanide were less likely to be admitted to an ICU (OR, 0.17; 95% CI, 0.04–0.75) vs. placebo (Figure 4; Supplementary Material). Additionally, when corticosteroids were given concomitantly with nitazoxanide, patients were less likely to be admitted to the ICU (OR, 0.45; 95% CI, 0.22–0.96) than placebo recipients who were given corticosteroids. Corticosteroid therapy alone did not affect the ICU admission rate (OR, 1.86; 95% CI, 0.97–3.57) (Figure 4; Supplementary Material).

## Discussion

Drug repurposing can reduce the drug development process and allow rapid deployment of effective therapies in a pandemic scenario. In this line, based on the antiviral and anti-inflammatory effects of nitazoxanide (7, 8, 10), combined with its wide availability, oral administration, and safety, our study evaluated the effects of this therapy in patients hospitalized with COVID-19 pneumonia. Nitazoxanide had no impact on ICU admission due to the need for invasive mechanical ventilation (the primary outcome). Nevertheless, nitazoxanide accelerated symptom resolution, shortened duration of oxygen therapy and reduced levels of inflammatory mediators (secondary outcomes). Due to the low cost and large availability of nitazoxanide, it might be considered in middle- and low-income countries to better control the spread of the pandemic.

The median recovery time was reduced by 1 day with nitazoxanide vs. placebo, which may be associated with decreased pro-inflammatory mediators. Kaplan-Meier time-to-event curves up to day 14 also suggested that clinical improvement and decreased requirement for supplemental oxygen led to earlier discharge in the nitazoxanide group compared with the placebo group. Beneficial effects were mostly observed between days 3 and 7; none were detected on day 14. This may be explained by the fact that nitazoxanide was administered from day 1 to day 5. Further studies are required to evaluate whether different results would be observed by extending the period of nitazoxanide administration. The frequency and severity of adverse events did not differ significantly between the groups, suggesting that nitazoxanide is safe for patients with COVID-19 pneumonia.

In patients with COVID-19 pneumonia undergoing nitazoxanide therapy, 54% of mITT population also received corticosteroids. Interestingly, these patients were less likely to be admitted to the ICU. In the placebo group (55% of mITT population) receiving corticosteroid therapy alone, ICU admission rate was not affected. In the RECOVERY trial (17), patients with COVID-19 pneumonia requiring supplementary oxygen or non-invasive mechanical ventilation treated with dexamethasone alone showed a reduced risk of progression to invasive mechanical ventilation and ICU admission. However, our study was not designed to evaluate corticosteroid therapy alone. Even though dexamethasone is available for patients with COVID-19 pneumonia; there are concerns regarding adverse reactions with its use, particularly reports on hospital-acquired infections, gastrointestinal bleeding, neuromuscular weakness, fungal infections, even with short courses (18–20).

In the mITT-positive population, nitazoxanide did not reduce the viral load, which was not attributed to the timing of hospital admission. However, patients with a higher viral load at hospital admission showed a trend toward viral load reduction (*p* = 0.054) in the nitazoxanide group vs. the placebo group.

In agreement with our findings, Blum et al. (10) described, in a proof-of-concept pilot trial, that nitazoxanide decreased the mean time to hospital discharge, and inflammatory mediators in hospitalized COVID-19 with mild respiratory insufficiency.

Our trial has several challenges: (1) only hospitalized patients in the ward were included. Because there were few beds available, patients remained in the emergency room. Therefore, it took 6 months to complete the study. (2) Online training and monitoring visits were made available to clinicians. (3) Personal protective equipment and some supplies were scarce at the beginning of the trial. (4) Some antivirals such as Remdesivir are not available in the public hospitals.

## Limitations and Strengths

This study has several limitations. Outcome ascertainment was limited to 14 days after randomization because most patients were discharged before this time point (only 5.9% remained in the hospital). This trial evaluated nitazoxanide as monotherapy for COVID-19, not associated with other antivirals or anti-inflammatory agents (other than corticosteroids after the publication of the RECOVERY trial). Secondary outcomes were underpowered because the study was designed based on the primary outcome; however, this study is a step forward for designing clinical trials to confirm the findings related to the secondary outcomes. The study started in April 2020 when the RT-PCR test was not easily available, therefore we opted to also add a CT scan as an inclusion criterion when cases were highly suggestive of SARS-CoV-2 pneumonia. Concerning the secondary outcomes, in the current study, we opted to focus mainly on respiratory parameters (e.g., respiratory rate, oxygen saturation).

The strengths of our study include the following: (1) randomized, double-blind, and prospective design, the large sample size of patients hospitalized with COVID-19 only in the ward, the enrolment of patients from different parts of Brazil, reflecting different patient characteristics, practice patterns, and health care systems; (2) analyses of ITT and mITT and mITT-positive population; (3) nitazoxanide did not prevent ICU admission; (4) nitazoxanide reduced the recovery time, thus improving clinical outcomes and decreasing the requirement for supplemental oxygen, which reduced the burden on the health care system, potentially increasing hospital capacity.

## Conclusions

In patients hospitalized with COVID-19 pneumonia, nitazoxanide, compared with placebo, did not prevent ICU admission, but may accelerate symptom resolution, shorten duration of oxygen therapy and reduce levels of inflammatory mediators. In addition, nitazoxanide demonstrates a good safety profile. Even though vaccination has increased worldwide, some countries are facing a fourth wave. Moreover, countries in Africa, Asia, and Latin America present numerous difficulties and require cheaper drugs to treat COVID-19 pneumonia.

## Data Availability Statement

The raw data supporting the conclusions of this article will be made available by the authors, without undue reservation.

## Ethics Statement

The studies involving human participants were reviewed and approved by Brazilian National Commission for Research Ethics (CAAE:30662420.0.1001.0008). The patients/participants provided their written informed consent to participate in this study.

## Author Contributions

PR, PS, and FC were involved in the conception and design of the study. PT, ER, JJ, FH, RÁ, JS, MM, KB, LB, AC, AJ, JA-F, LC, PA, AM, RC, MO, RS, and CS were responsible for recruitment and clinical care of the patients. AF was responsible for laboratory analyses. KF, PP, and JL-e-S contributed to data interpretation and critical review of the manuscript. RL, SM, CS, and CM were responsible for the statistical analysis. NF, PM-S, CN, and DC contributed to data collection. All authors reviewed and approved the final version of the manuscript.

## Funding

This work was supported by the Brazilian Council for Scientific and Technological Development (CNPq), Brazilian Ministry of Science, Technology, and Innovation for Virus Network; Brasília, Brazil (number: 403485/2020-7), and Funding Authority for Studies and Projects (FINEP), Brasília, Brazil (number: 01.20.0003.00).

## Conflict of Interest

CM was employeed by AAC&T Research Consulting LTDA. The remaining authors declare that the research was conducted in the absence of any commercial or financial relationships that could be construed as a potential conflict of interest.

## Publisher's Note

All claims expressed in this article are solely those of the authors and do not necessarily represent those of their affiliated organizations, or those of the publisher, the editors and the reviewers. Any product that may be evaluated in this article, or claim that may be made by its manufacturer, is not guaranteed or endorsed by the publisher.
