# Supplementary material for: Nitazoxanide in Patients Hospitalized With COVID-19 Pneumonia: A Multicentre, Randomized, Double-Blind, Placebo-Controlled Trial
Source: Front Med (Lausanne). 2022 Apr 13;9:844728. doi: 10.3389/fmed.2022.844728 (PMC9043450; doi:10.3389/fmed.2022.844728)
Supplement: Supplementary file 1 [file Data_Sheet_1.DOCX]

**Nitazoxanide** **in** **patients hospitalized with COVID-19 pneumonia: a multicentre, randomized, double-blind, placebo-controlled trial**

**Supplementary Material 1**

**SARITA-1 Collaborative Group**

**Table of Contents:**

| **Committees, Leadership, and Investigators** | 3 |
| --- | --- |
| **Supplementary Methods** | 8 |
| Study organization | 8 |
| Protocol changes | 8 |
| Study population | 8 |
| Supplementary statistical methods | 10 |
| Supplementary details on the randomization procedure | 10 |
| Supplementary details on data collection | 10 |
| Supplementary details on interventions | 11 |
| Supplementary details on outcomes | 12 |
| Supplementary details on monitoring | 13 |
| **Supplementary Tables** |  |
| SMTable 1. List of sites and number of randomized patients per site | 14 |
| SMTable 2. Baseline characteristics of the ITT COVID-19 population | 15 |
| SMTable 3. Baseline characteristics of the mITT-positive COVID-19 population | 20 |
| SMTable 4. Comparison of baseline characteristics between the ITT, mITT, and mITT-positive COVID-19 populations | 23 |
| SMTable 5. Comparison of the primary outcome between the mITT, ITT, and mITT-positive populations | 25 |
| SMTable 6. Eight-point ordinal scale of clinical status measured daily in the mITT population treated with nitazoxanide or placebo until day 14 | 26 |
| SMTable 7. Supplemental oxygen therapy requirement in the mITT population | 29 |
| SMTable 8. COVID-19 symptoms in the nitazoxanide and placebo groups at different time points | 31 |
| SMTable 9. Medications received during the study in the mITT population | 33 |
| SMTable 10. Viral load and RT-PCR status in the ITT, mITT, and mITT-positive populations | 34 |
| SMTable 11. Nasopharyngeal swab RT-PCR viral load stratified according to its level on day 1 (baseline) | 35 |
| SMTable 12. Secondary outcomes in the mITT population | 36 |
| SMTable 13. Abnormal findings on chest CT scans in the mITT population | 38 |
| SMTable 14. Adverse events in the ITT population | 39 |
| SMTable 15. Comparison of primary outcome occurrence between selected characteristics, independent of treatment | 40 |
| **Supplementary Figures** |  |
| SMFigure 1. Categories on an eight-point ordinal scale in the nitazoxanide and placebo groups at different time points | 42 |
| SMFigure 2. Oxygen requirement in the nitazoxanide and placebo groups at different time points | 43 |
| SMFigure 3. COVID-19 symptoms in the nitazoxanide and placebo groups at different time points | 45 |
| **References** | 46 |

**Details of the SARITA-1 Collaborative Group**

**Committees, Leadership, and Investigators**

**SARITA-1**: The SARITA-1 trial is supported by the Brazilian Ministry of Science, Technology, and Innovation (MCTI), formerly the Ministry of Science, Technology, Innovation, and Communications (MCTIC), and coordinated by the Federal University of Rio de Janeiro (lead investigator: Prof. Patricia R.M. Rocco)

**Executive Committee:** Patricia R. M. Rocco, M.D., Ph.D.,^1^ Pedro L. Silva, Ph.D.,^1^ Fernanda F. Cruz, M.D., Ph.D.^1^

**Steering Committee:** Patricia Rieken Macedo Rocco, M.D., Ph.D.^1^; Pedro Leme Silva, Ph.D.^1^; Fernanda Ferreira Cruz, M.D., Ph.D.^1^; Paulo Fernando Guimarães Morando Marzocchi Tierno, M.D.^2^; Eucir Rabello, M.D.^3^, M.SC.; Jéfiton Cordeiro Junior, M.D.^4^; Firmino Haag Ferreira Junior, M.D.^5^; Renata Eliane de Ávila, M.D., M.Sc., Ph.D.^6^; Joana Darc Gonçalves da Silva, M.D., M.Sc.^7^; Mariana Márcia Santos Mamede, M.Sc., Ph.D.^8^; Konrad Silva Buchele, M.D.^9^; Luiz Carlos Viana Barbosa, M.D.^10^; Anna Caryna Cabral, M.D., Ph.D.^11^; Antonio Augusto Freitas Junqueira, M.D.^12^; João Alves Araújo Filho, M.D., Ph.D.^13^; Lucianna Auxi Teixeira Josino da Costa, M.D., M.Sc.^14^; Pedro Paulo Martins Alvarenga, M.D., M.SC.^15^; Alexandre Sampaio Moura, M.D., MPH, Ph.D.^16^; Ricardo Carajeleascow, M.D.^17^; Mirella Cristine de Oliveira, M.D.^18^; Roberta Gunnutzmann Ferreira Silva, M.D.^19^; Cynthia Regina Pedrosa Soares, M.Sc.^20^; Ana Paula Salles Moura Fernandes, Ph.D. ^21^; Flávio Guimarães da Fonseca, Ph.D.^21^; Vidyleison Camargos, Ph.D.^21^; Julia Reis, Ph.D.^21^; Kleber Franchini, M.D., Ph.D.^22^, Ronir R. Luiz, Ph.D.^1^; Sirlei Morais, PhD^23^; Carlos Sverdloff, Pharm.D., M.Sc.^23^; Camila Marinelli Martins, Ph.D,^24^; Nathane Santanna Felix, M.D., Ph.D. ^1^; Paula Mattos Silva, Ph.D.^1^; Caroline Muiler Barbosa Nogueira, M.Sc^1^; Dayene de Assis Fernandes Caldeira, M.Sc.^1^; Paolo Pelosi, M.D., FERS,^25, 26^ José Roberto Lapa e Silva, M.D., Ph.D.^1^; on behalf of the SARITA-1 investigators.

**Affiliations:**

1. Federal University of Rio de Janeiro, Rio de Janeiro, Rio de Janeiro, Brazil

2. Hospital Municipal de Barueri Dr Francisco Moran, Barueri, São Paulo, Brazil

3. Hospital da Força Aérea do Galeão, Rio de Janeiro, Rio de Janeiro, Brazil

4. Hospital Regional de Sorocaba Dr Adib D Jatene- Bata Branca, Sorocaba, São Paulo, Brazil

5. Hospital Geral de São Mateus – Dr. Manoel Bifulco, São Mateus, São Paulo, Brazil

6. Hospital Eduardo Menezes, Belo Horizonte, Minas Gerais, Brazil

7. Hospital Regional da Asa Norte, Brasília, Distrito Federal, Brazil

8. Hospital das Forças Armadas, Brasília, Distrito Federal, Brazil

9. Hospital Naval Marcilio Dias, Rio de Janeiro, Rio de Janeiro, Brazil

10. Hospital das Clínicas Luzia de Pinho Melo, Mogi das Cruzes, São Paulo, Brazil

11. Hospital Universitário Pedro Ernesto, Rio de Janeiro, Rio de Janeiro, Brazil

12. Hospital Central da Aeronáutica, Rio de Janeiro, Rio de Janeiro, Brazil

13. Hospital Estadual de Doenças Tropicais Dr. Anuar Auad, Anápolis, Goiás, Brazil

14. Hospital Geral de Fortaleza, Fortaleza, Ceará, Brazil

15. Hospital Mater Dei, Belo Horizonte, Minas Gerais, Brazil

16. Santa Casa de Misericórdia de Belo Horizonte, Belo Horizonte, Minas Gerais, Brazil

17. Complexo Hospitalar Municipal de São Caetano do Sul, São Caetano do Sul, São Paulo, Brazil

18. Complexo do Trabalhador de Curitiba, Curitiba, Paraná, Brazil

19. Hospital da Força Aérea de São Paulo, São Paulo, São Paulo, Brazil

20. Hospital das Clínicas da Universidade Federal do Pernambuco, Recife, Pernambuco, Brazil

21. Centro de Tecnologia de Vacinas, Universidade Federal de Minas Gerais, Belo Horizonte, Minas Gerais, Brazil

22. State University of Campinas, Campinas, São Paulo Brazil

23. ATCGEN, Campinas, São Paulo, Brazil

24. AAE&T Research Consulting, Curitiba, Paraná, Brazil

25. Department of Surgical Sciences and Integrated Diagnostics, University of Genoa, Genoa, Italy

26. San Martino Policlinico Hospital, IRCCS for Oncology and Neurosciences, Genoa, Italy

**Data and Safety Monitoring Board (DSMB)**

Prof. Marcus Conte, M.D., Ph.D.^1^; Prof. Hugo Caire Faria Neto, M.D., Ph.D.^2^; Prof. Hugo Oliveira, M.D., Ph.D..^3^

**Affiliations:**

1. University of Petrópolis, Petrópolis, Rio de Janeiro, Brazil

2. FIOCRUZ, Rio de Janeiro, Rio de Janeiro, Brazil

3. Federal University of Rio Grande do Sul, Porto Alegre, Rio Grande do Sul, Brazil

**Local Clinical Centre SARITA-1 Trial Staff**

All trial sites are located in Brazil and listed in descending order according to the number of randomized patients per site.

**Hospital Municipal de Barueri Dr Francisco Moran, Barueri, São Paulo, Brazil**

Paulo Fernando Guimarães Morando Marzocchi Tierno, Nara Franzin de Moraes, Simone Luna de Almeida, Ana Paula Silva Araújo, Michelle Cardoso Billet Miranda, Denilda de Jesus Ferreira, Eliana Pereira da Silva, Andreia Avelino Oliveira, Henilton Alves Tonelli, Fernando Carvalho Sacchetin, Igor Silva Fernandes Machado, Ana Lúcia dos Santos Silva

**Hospital da Força Aérea do Galeão, Rio de Janeiro, RJ, Brazil**

Eucir Rabello, Adriana Ouverney Braz, Marcelo Rolla de Souza, Antonio Arnaldo de Carvalho Machado Neto, Ana Cristina Ferreira de Souza, Cidcley Samia, Raphael Paiva Paschoal da Silva, Emmanuella Andrade Leal, Heitor Castro Júnior, Raissa Maria Lapa; Patrícia Soares de Jesus

**Hospital Regional de Sorocaba Dr Adib D Jatene- Sorocaba, São Paulo, Brazil**

Jéfiton Cordeiro Junior, Luara Piovan Garcia Mello Ribeiro, Thaís Picao Batista, Flávia Aparecida da Costa, Marcelo Bueno de Camargo, Gleice Santos da Silva

**Hospital Geral de São Mateus – Dr. Manoel Bifulco – São Mateus, São Paulo, Brazil**

Firmino Haag Ferreira Junior, Alessandra Castilho Mansano Sanches, Victor Mendes Leal Costa, Sandra Maria Barroso Bastos Silveira, Diany Priscila Silva de Oliveira, Edesio Vieira da Silva Filho

**Hospital Eduardo Menezes- Fundação Hospitalar do Estado de Minas Gerais, Belo Horizonte, Minas Gerais, Brazil**

Renata Eliane de Ávila, Maria Candeias, Tássia Lopardi Pereira, Tayrine Araújo, Vinícius Gonçalves de Paulo

**Hospital Regional da Asa Norte, Brasília, Distrito Federal, Brazil**

Joana Darc Gonçalves da Silva, Ulysses Rodrigues de Castro, Ana Helena Britto Germoglio, Wendel dos Santos Furtado, Tamiris Alves Menezes, Mariana Cabral Silva, Gilson Roberto de Araújo, Paulo Henrique Ramos Feitosa, Ana Katarina da Silva Santos, Brunna Machado Barbosa, Nathane Carolina Vieira de Sales, Antônia Arnóbia Viana Lima e Azambuja

**Hospital das Forças Armadas, Brasília, Distrito Federal, Brazil**

Mariana Márcia Santos Mamede, Aline Mizusaki Imoto, Hemerson dos Santos Luz, Claudia da Matta Corrêa, Claudia Rocha Dias, Rosangela de Sousa Martins, Rosana Leite Trojan, Marcio de Campos e Silva, Daniela Boneberger BEHm, Lucas Crespilho da Silva, Fábio Cardoso Reis, Clarissa Bárbara Queiroz da Silva, Larissa Prado de Souza Carvalho, Gustavo Martins Gomes Fontoura, Ravla Faria Pereira da Silva, Thiago Hayashida Teles de Carvalho, Emanuel Acaiaba Reis de Sousa Filho, Heloísa de Lima Soares, Maria Livia Daniela Ribeiro Barbosa, Silvia Porphirio Orioli Caldas, Adenalva Lima de Souza Beck, Scheila Gomes Cardoso Boeira.

**Hospital Naval Marcilio Dias, Rio de Janeiro, RJ, Brazil**

Konrad Silva Buchele, Laís Vilanova Tavares Vitoriano, Marcelo Leal Gregório, André Germano De Lorenzi, Romero José de Carvalho Júnior, Aristides Bezerra de Melo Junior, Laís Vilanova Tavares Vitoriano

**Hospital das Clínicas Luzia de Pinho Melo, Mogi das Cruzes, São Paulo, Brazil**

Luiz Carlos Viana Barbosa, Roseli Gomes Cavalini, Iraci Ferreira Xavier, Faustino Peron Filho, Gustavo Bittencourt dos Santos, Luciana Pontes dos Santos, Roselane Pereira Lino da Silva, Erika Frutuosa dos Santos Souza, Maria Eclisiane Alves Lima

**Hospital Universitário Pedro Ernesto, Rio de Janeiro, RJ, Brazil**

Anna Caryna Cabral, Marcio Neves Bóia, Natan de Araújo Aragão, Graziela Machado Baptista, Rui Teófilo Figueiredo Filho, Gustavo Adolfo Brasileiro Passos, Barbara Hasselmann Fernandes de Oliveira, Lúcia Helena Cavalheiro Villela, Caio César de Oliveira Rocha, Ana Lúcia Nascimento de Oliveira

**Hospital Central da Aeronáutica, Rio de Janeiro, RJ, Brazil**

Antonio Augusto Freitas Junqueira, Arilson Dias, Fabio Basilio Fernandes dos Santos, Alexandre Moraes Ferreira, Bruna Rocha da Silva, Luiggi Miguez Dantas, Aloisio Batista Pereira, Beatriz Antunes Pereira, Gabriele Borba, Vanessa Cruz Malizia, Cláudia Pellegrino Freire, Pedro Luiz Naglis Tiburcio, Leomar Gomes Moreira Santos, Jazon Benine Leal, Camila Carvalho Lopes, Alyne Freitas Pereira Gondar, Meryellen Lopes Basílio, Isabela Azevedo Mota, Alan Igor Herzog Mação Campos, Michele Lúcia de Aguiar Mitsuyasu, Izabela Rosa Gonçalves Montoaneli, Diogo da Silva, Leandro Figueira Reis de Sá, André Lassance de Oliveira Alonso Martinez, Fernanda Mesquita Abi-Rihan Cordeiro, Lione da Silva, Camila Aguiar Lomônaco, Kelly Rafael Pinto Gomes, Rafael Machado de Oliveira Lima, Matheus Da Silva Tavares Henriques, Djanine de Paula Nascimento

**Hospital Estadual de Doenças Tropicais Dr. Anuar Auad, Goiânia, Goiás, Brazil**

João Alves Araújo Filho, Renata de Bastos Ascenço Soares, Mirela Pereira Lima, Cristielly Guimarães Franco, Fernanda Pedrosa Torres, Luiz Alves da Silva Neto, Taiguara Fraga Guimarães

**Hospital Geral de Fortaleza, Fortaleza, Ceará, Brazil**

Lucianna Auxi Teixeira Josino da Costa, Patrícia Amanda Pereira Vieira, Maria de Lourdes Aguiar da Silva Sousa, Isis Eduarda Caetano Batista Fontenele, Thaissa Pinto de Melo, Larissa Taumaturgo Baltazar

**Hospital Mater Dei, Belo Horizonte, Minas Gerais, Brazil**

Pedro Paulo Martins Alvarenga, Anselmo Dornas Moura, Alice Martins Alvarenga, Hugo Martins Moura, Caio Drumond Duarte, Thomas Zenobio Cruz, Luiza Barroso de Andrade, Daniela de Miranda Carneiro

**Santa Casa de Misericórdia de Belo Horizonte, Belo Horizonte, Minas Gerais, Brazil**

Alexandre Sampaio Moura, Isabela Olegário Bernardes Louredo, Claudio Dornas de Oliveira, Jomar Cristeli Nepomuceno, Yuri José Ferreira da Conceição, Renan Pazini Borges, Ariadne Poliana de Oliveira Bastos

**Complexo Hospitalar Municipal de São Caetano do Sul, São Caetano do Sul, São Paulo, Brazil**

Ricardo Carajeleascow, José Antonio Manetta, Ana Lívia Bellini de Rizzo de Figueiredo, Lídia Bruna Frassato Movio, Francyone da Silva

**Complexo do Trabalhador de Curitiba, Curitiba, Paraná, Brazil**

Mirella Cristine de Oliveira, Mariana Bruinje Cosentino, Fernanda Baeumle Reese, Bruno Alcântara Gabardo, Márcio P. Ribeiro;

**Hospital da Força Aérea de São Paulo, São Paulo, São Paulo, Brazil**

Roberta Gunnutzmann Ferreira Silva, Laura Maria Fontes Prado Moraes, Fernando Mallet Soares Paragó, Fernanda Aparecida Gonçalves

**Hospital das Clínicas da Universidade Federal do Pernambuco, Recife, Pernambuco, Brasil**

Cynthia Regina Pedrosa Soares, Paulo Sérgio Ramos de Araújo, Valdemir Cordeiro de Paula

**Coordinating Centre SARITA-1 Staff**

**Federal University of Rio de Janeiro, Rio de Janeiro, Brazil**

Patricia Rieken Macedo Rocco, Pedro Leme Silva, Fernanda Ferreira Cruz, Dayene de Assis Fernandes Caldeira, Nathane Santana Félix, Caroline Muiler Barbosa Nogueira, Paula de Mattos Silva, André Benedito da Silva, Heloísa Leal Setta, Ronir Raggio Luiz, José Roberto Lapa e Silva;

**RT-PCR Laboratory Centre SARITA-1 Staff**

**Centro de Tecnologia de Vacinas, Universidade Federal de Minas Gerais, Belo Horizonte, Minas Gerais, Brazil**

Ana Paula Fernandes, Alex Fiorini de Carvalho, Raissa Prado Rocha, Vidyleison Neves Camargos, Flávio Guimarães da Fonseca, Helena Perez Coelho, Júlia de Souza Reis

**Laboratório de Imunologia de Doenças Virais, Instituto René Rachou, Fiocruz, Belo Horizonte, Minas Gerais, Brazil**

Pedro Augusto Alves

**Instituto Adolfo Lutz:** Carlos Roberto Prudêncio

**Monitoring Service SARITA-1 Staff**

**ATCGen, Campinas, São Paulo, Brazil**

Sirlei Morais, Carlos Sverdloff

**Manufacturer Representatives SARITA-1 Staff**

**Eurofarma**

Cassiano Ricardo de Oliveira Berto, Pharm.D.; Walker Magalhães Lahmann, M.D.

**Information Technology Team**

**Military Institute of Engineering (IME)**

Marcelo Mendes da Silveira, Leandro Feijó Breves, Lucas Sebastião de Paula, Wallace Andrade da Silva, Anderson Fagundes Santos.

**Statistical Support SARITA-1 Staff**

**AAE&T Research Consulting**

Camila Marinelli Martins

**ATCGen, Campinas, São Paulo, Brazil**

Sirlei Morais

**Federal University of Rio de Janeiro**

Ronir R Luiz**Supplementary Methods**

**Study Organization**

SARITA-1 is a multicenter, randomized, placebo-controlled, parallel, double-blinded, interventional, treatment clinical trial with two arms, which aims to study the impact of nitazoxanide for patients hospitalized with pneumonia derived from COVID-19 (Coronavirus Disease-19). The protocol is available. The trial was conducted at 19 hospital organizations in Brazil and was coordinated by a team drawn from the Federal University of Rio de Janeiro, sponsored by the Brazilian Council for Scientific and Technological Development (CNPq), Brazilian Ministry of Science, Technology, and Innovation for Virus Network; Brasília, Brazil (number: 403485/2020-7) and Funding Authority for Studies and Projects (FINEP), Brasília, Brazil (number: 01.20.0003.00). The trial followed the principles of the Declaration of Helsinki and the International Conference on Harmonization (ICH) Good Clinical Practice Guideline (E6R2). Online clinical monitoring and quality control were outsourced to a contract research organization (ATCGen, Campinas, Brazil).

Nitazoxanide was provided free of charge by Eurofarma (São Paulo, Brazil), which had no other role in the design or conduct of the trial. The executive committee assures the accuracy of the data and fidelity of the trial to the protocol. The protocol was registered in the Brazilian Registry of Clinical Trials (REBEC) number RBR-88bs9x and ClinicalTrials.gov number NCT04561219. The independent Data and Safety Monitoring Board (DSMB), composed of experts in clinical trials and infectious diseases, was convened after 25%, 50%, and 75% of the participants had completed 14 days of follow-up and had access to information on adverse events and efficacy outcomes at every quartile.

**Protocol Changes**

SARITA-1 is a randomized placebo-controlled trial among patients hospitalized for COVID-19. All eligible patients receive usual standard of care in the participating hospital and are randomly allocated between treatment with either nitazoxanide or placebo. Over time, additional hospitals have been added (see Protocol SARITA-1).

The original and final protocols are included in the protocol annexed to this publication, together with summaries of the changes made.

**Eligible Study Population**

Consecutive adult patients (aged 18 years or older) requiring supplemental oxygen (peripheral oxygen saturation [SpO_2_] <93%) were admitted to hospital with COVID-19 symptoms (fever, dry cough, productive cough, shortness of breath, sore throat) associated with chest computed tomography (CT) scan findings suggestive of viral pneumonia or positive nasopharyngeal swab test for SARS-CoV2 (reverse-transcriptase polymerase chain reaction [RT-PCR]). Due to the delay in SARS-CoV-2 testing early in the pandemic, the trial was initially designed to enroll hospitalized patients with suspected or confirmed SARS-CoV-2 infection. Written informed consent was obtained from all patients or from a legal representative if they were unable to provide consent. A nasopharyngeal swab was then collected for RT-PCR testing, regardless of a previous positive SARS-CoV-2 test. To mitigate any bias, all RT-PCR analyses were processed centrally at CT-VACINAS, Federal University of Minas Gerais, Belo Horizonte, Brazil; specimens were sent on the day of collection by commercial courier. Patients discharged from the hospital before day 5 continued the trial medication to complete the 5-day course and were asked to return on days 7 and 14 for clinical and laboratory analyses. Patients who did not return to the study sites were contacted by telephone to understand the reasons for nonadherence to the study protocol.

***Inclusion criteria***

1. Symptoms of SARS-CoV-2 infection associated with a chest CT scan suggestive of viral pneumonia, SpO_2_ <93%;

2. Symptoms of SARS-CoV-2 infection associated with a positive nasopharyngeal swab test for SARS-CoV2 (RT-PCR), SpO_2_ <93%;

3. Age ≥18 years, male or female;

4. Negative pregnancy test;

5. Willingness to take the study therapy;

6. Provision of written informed consent (by patient or a health care surrogate).

***Exclusion criteria***

1. Inability to swallow;

2. History of severe liver disease;

3. Chronic kidney disease defined as an estimated glomerular filtration rate of less than 30 mL/min/1.73 m^2^;

4. Severe heart failure (NYHA class 3 and class 4);

5. Severe chronic obstructive pulmonary disease (COPD) (GOLD 3 and 4);

6. Any cancer in the last 5 years;

7. Any known autoimmune disease;

8. Known allergy to nitazoxanide or its components;

9. Nitazoxanide treatment in the last 30 days;

10. Clinical suspicion of tuberculosis or bacterial pneumonia.

**Eight-point ordinal clinical scale**

The 8-point ordinal clinical scale consisted of the following categories: (1) not hospitalized, no limitation of activities; (2) not hospitalized, limitation of activities; (3) hospitalized, not requiring supplemental oxygen or ongoing medical care (used if hospitalization was extended for infection-control reasons); (4) hospitalized, not receiving supplemental oxygen, but requiring ongoing medical care (COVID-19–related or other medical conditions); (5) hospitalized, receiving supplemental oxygen; (6) hospitalized, receiving nasal high-flow oxygen or noninvasive mechanical ventilation; (7) hospitalized, receiving invasive mechanical ventilation or extracorporeal membrane oxygenation (ECMO); and (8) death.

**Supplementary Statistical Methods**

***Sample size calculation***

The sample size calculation was based on the assumption that around 15% of hospitalized patients with COVID-19 were admitted to the intensive care unit (ICU) in March 2020. Assuming that 5% of hospitalized patients treated with nitazoxanide would be admitted to the ICU, with an alpha error of 5%, statistical power of 90%, and two-tailed test, 198 participants per group would then be required. Considering an estimated dropout rate of about 20%, our target sample size was set at 247 participants/group (intention-to-treat population [ITT]). The sample size calculation was done using G*Power 3.1.9.2 (University of Düsseldorf, Düsseldorf, Germany).

***Statistical plan***

Data will be analyzed according to distribution, whether parametric or non-parametric. Parametric data will be expressed as mean ± standard deviation (SD) and non-parametric data as median (interquartile range). At the end of 7 and 14 days, Student's t test, Mann-Whitney U test, Chi-square test, or Fisher's exact test will be used to compare differences between those with and without respiratory failure. Univariate and multivariate logistic regression models will be used to recognize the risk factors associated with hospital mortality. Significance will be displayed as *P* < .05.

**Supplementary Details on the Randomization Procedure**

The trial statistician, not involved with patient enrollment or care, obtained a computer-generated randomization list (random.org). The Information Technology Team inserted the list on the secure website. Participants were randomized (1:1 ratio) using this list to either the control arm (group B, placebo) or the intervention arm (group A, nitazoxanide). The study treatment (A or B) was revealed to the pharmacist only after patients were registered in the system, ensuring proper concealment of the allocation sequence. The designated pharmacist at each study site was the only person aware of group allocation throughout the trial.

**Supplementary Details on Data Collection**

A secure website was created by the Information Technology Team (see Committees, Leadership, and Investigators) for data entry, validation, collection, and export. Site investigators, ATCGen monitors, and executive committee members were assigned a secure login and encrypted passwords (128-bit hash). The SARITA-1 system was built on PHP with an mySQL database as the general system; PHP as the web layer; DDD architecture with dependency injection and control inversion as the backend; jQuery with Bootstrap as the front end. The system was hosted on Amazon Webservice. After patient registration in the secure website, electronic case reports from the system (REDCap and clinical trial management system-ATCGen) were sent by specific monitors on a daily basis, according to data gathered by each site investigator and further validated by external trial monitoring staff at ATCGen.

Upon registration of a patient in the system, a unique trial identifier and barcode number (for laboratory tests) were generated, and the patient was randomly allocated to group A or B, as mentioned above. Forms within the system were divided into sections that allowed registration of (1) personal data: contact information, patient demographic data, comorbidities, medication use, general comments; and upload of informed consent forms; (2) clinical data: vital signs, symptoms, clinical status, clinical evolution, oxygen need, and adverse events daily until day 7, and on day 14; (3) nasopharyngeal swab collection data: viral load by RT-PCR at baseline and on day 7; (4) complete blood cell count data daily until day 7 and on day 14; (5) laboratory results: C-reactive protein, D-dimer, ferritin, dehydrogenase lactate at baseline and days 3 and 7; (5) radiological information (chest CT scan findings) at baseline, and days 3 and 7.

**Supplementary Details on Interventions**

***Timeline***

When eligible, patients were randomized as mentioned above. On day 1, baseline blood samples were collected, and clinical evaluation was done before treatment began. In addition, a chest CT scan was performed, and a nasopharyngeal swab was collected from patients for molecular confirmation of SARS-CoV-2 infection by RT-PCR. If the chest CT scan suggested COVID-19 pneumonia, patients were included in the study. If a chest CT scan was unavailable or did not suggest COVID-19 changes, patient inclusion depended on the RT-PCR test. If the chest CT scan and RT-PCR were negative, patients were not included in the study. Patients were randomized to receive treatment with either nitazoxanide 500 mg or placebo every 8 hours for 5 days according to group allocation.

Every patient included in the study was monitored according to the following data collection plan:

1. Clinical evaluation (vital signs, systemic and respiratory symptoms, oxygen support level and type, clinical complications, adverse events, concomitant medications): daily until day 7 and on day 14;

2. Nasopharyngeal swab collection for viral load assessment by RT-PCR at baseline and on day 7;

3. Chest CT scan at baseline and on days 3 and 7;

4. Complete blood cell count daily until day 7 and on day 14;

5. C-reactive protein, D-dimer, ferritin, dehydrogenase lactate, troponin, electrolytes, urea, creatinine, glucose, coagulation tests at baseline and on days 3 and 7.

***RNA extraction and real-time polymerase chain reaction***

Nasopharyngeal swab samples obtained from each patient were collected in a single tube containing 2 mL of guanidine isothiocyanate transport solution. Extraction of the total RNA from collected specimens was performed using the QIAamp Viral RNA Mini Kit (QIAGEN, USA), following the manufacturer’s protocols. Quantitation of viral RNA was performed by quantitative RT-PCR following the Berlin (Charité) protocol, using the Bio Gene Covid-19 PCR kit (Bioclin, Brazil) according to the manufacturer’s instructions (Rocco et al., 20. The quantitative RT-PCR reaction was performed in a QuantStudio 3 or QuantStudio 5 Real-Time PCR System (Thermo Fisher, USA). Human RNase P mRNA was used as internal control and to correct the SARS-CoV-2 viral load in each sample by adjusting viral gene Ct values; to correct the Ct value of SARS-CoV-2 E gene amplification of each sample, Ct values were normalized using the following equation: (sample SARS-CoV-2 E Ct value × sample RNaseP Ct value/plate mean RNaseP Ct value). Standard curves were produced by using serial 10-fold dilutions of standard synthetic RNA transcripts of SARS-CoV-2 E gene, ranging from 2 to 2×10^5^ copies/µL (Bioclin, Brazil). Absolute quantification of the genomic viral load was performed by comparing sample Ct values with the standard curve. All samples were evaluated centrally at a single site (Centro de Tecnologia de Vacinas, Universidade Federal de Minas Gerais, Brazil).

***Chest computed tomography scan***

Chest CT scans were done at baseline and on days 3 and 7. The major CT findings were described using international standard nomenclature defined by the Fleischner Society glossary and peer-reviewed literature on viral pneumonia, using terms including ground-glass opacity, crazy-paving pattern, pleural effusion, and consolidation (Hansel et al., 2008, Franquet, 2011). A semiquantitative scoring system was used to estimate the pulmonary involvement of all these abnormalities on the basis of the area involved: 0%–25%, 26%–50%, 51%–75%, and 75%–100% involvement.

***Complete blood counts and laboratory mediator quantification***

Every study site collected blood from patients at different time points mentioned above. Complete blood cell count and the other parameters were analyzed at the local laboratory of each hospital. Cryotubes were labeled with the patient’s unique trial identifier and the date of specimen collection.

**Supplementary Details on Outcomes**

This study evaluated efficacy and safety outcomes.

***Primary outcomes***

The primary outcome was ICU admission due to invasive mechanical ventilation at any point during patient hospitalization until day 14.

***Secondary outcomes***

Clinical status was assessed daily according to an 8-point ordinal scale until day 14 (Kalil et al., 2021) with the following categories: (1) not hospitalized, no limitation of activities; (2) not hospitalized, limitation of activities; (3) hospitalized, not requiring supplemental oxygen or ongoing medical care (used if hospitalization was extended for infection-control reasons); (4) hospitalized, not receiving supplemental oxygen, but requiring ongoing medical care (COVID-19–related or other medical conditions); (5) hospitalized, receiving supplemental oxygen; (6) hospitalized, receiving nasal high-flow oxygen or noninvasive mechanical ventilation; (7) hospitalized, receiving invasive mechanical ventilation or ECMO; and (8) death. Time to recovery was defined as the first day up to day 14 of follow-up when the patient was discharged.

Secondary outcomes also included: (1) the proportion of patients with clinical improvement (time to improve two categories on an 8-point ordinal scale of clinical status at baseline [day 1]) on days 3, 5, 7, and 14; (2) the number of patients discharged (ordinal scale 1–2); (3) oxygen requirement (ordinal scale 5–6) (1 L O_2_/min, 1–5 L O_2_/min, high-flow oxygen through a nasal cannula, and invasive mechanical ventilation); (4) the number of patients who died (ordinal scale 8) from randomization until day 14; (5) symptom duration (dry and productive cough, sore throat, and shortness of breath) from baseline until day 14; (6) number of patients with negative RT-PCR on day 7 and reduction in viral RNA load on nasopharyngeal swab specimens (from baseline until day 7); (7) clinical (body temperature, respiratory rate, and SpO_2_) and laboratory data (hemoglobin levels, leucocytes, neutrophils, lymphocytes, platelets, C-reactive protein, D-dimer, ferritin, lactate dehydrogenase); and (8) chest CT scan score (0%–25%, 26%–50%, 51%–75%, and 76%–100%) at days 1, 3, and 7. All CT scan images were analyzed and reviewed by radiologists.

***Safety outcomes***

1. Incidence of adverse events (AEs) throughout the study;

2. Rate of treatment discontinuation due to AEs.

All outcomes were assessed by blinded investigators. We conducted source data verification of the day 14 assessment from the study sites and laboratory forms for all patients at the sites.

**Supplementary Details on Quality Monitoring**

Data quality monitoring was performed in accordance with the study monitoring plan. This monitoring was performed independently of the site investigators by trained dedicated staff from ATCGEN, a specialized contracted research organization in Brazil. This monitoring plan was elaborated in collaboration with the statistical team and the data managers from ATCGEN according to the protocol and the expected risks for patients. Data quality monitoring included remote data monitoring. During the main phase of the pandemic in Brazil, study monitors were not allowed to go on site. The status of electronic case report forms was reviewed remotely via web access to ensure that consents were valid, forms were being completed according to instructions, and queries were being resolved correctly. A predefined set of consistency checks was reviewed by the data manager of the clinical research unit and by the statistical team in an attempt to further validate the data and the queries that were raised directly on the study database. Data quality monitoring was performed secondarily by remote monitoring.

All “consent” and “consent withdrawn” documents were verified to ensure these were completed in accordance with the requirements approved by the ethics committee and, if consent was withdrawn, that this was documented appropriately. The presence of inclusion criteria and the absence of exclusion criteria at randomization, as well as correct measurement of the primary outcome, were verified. The 8-point ordinal scale scores were checked for all days between day 1 (baseline), day 7, and day 14, the type, start and stop dates of oxygen support (no oxygen requirement, 1 L O_2_/min, 1–5 L O_2_/min, high-flow oxygen through a nasal cannula, and invasive mechanical ventilation), presence of symptoms and resolution, radiological and laboratorial data, death dates and causes, and discharges dates. Verification was also performed on the relevant sections of the case report form for any trial participants for whom serious AEs were reported. In addition, the following sections of the case report form were also verified for 100% of patients at each site: (1) baseline form: comorbidities, treatments received at baseline, vital signs; (2) daily data form: all clinical and oxygen-related variables; (3) discharge and death form: ICU and hospital discharge date and time; (4) adverse event form: all questions on the form; (5) protocol violations for the nitazoxanide therapeutic scheme; (6) concomitant treatments received.

**SMTable 1. List of Sites and Number of Randomized Patients Per Site**

|  | **Hospital** | **Number of patients** |
| --- | --- | --- |
| **1** | Hospital Municipal de Barueri Dr Francisco Moran, Barueri, São Paulo, Brazil | 111 |
| **2** | Hospital da Força Aérea do Galeão, Rio de Janeiro, Rio de Janeiro, Brazil | 64 |
| **3** | Hospital Regional de Sorocaba Dr Adib D Jatene- Bata Branca, Sorocaba, São Paulo, Brazil | 51 |
| **4** | Hospital Geral de São Mateus – Dr. Manoel Bifulco, São Mateus, São Paulo, Brazil | 46 |
| **5** | Hospital Eduardo Menezes, Belo Horizonte, Minas Gerais, Brazil | 36 |
| **6** | Hospital Regional da Asa Norte, Brasília, Distrito Federal, Brazil | 31 |
| **7** | Hospital das Forças Armadas, Brasília, Distrito Federal, Brazil | 27 |
| **8** | Hospital Naval Marcilio Dias, Rio de Janeiro, Rio de Janeiro, Brazil | 24 |
| **9** | Hospital das Clínicas Luzia de Pinho Melo, Mogi das Cruzes, São Paulo, Brazil | 24 |
| **10** | Hospital Universitário Pedro Ernesto, Rio de Janeiro, Rio de Janeiro, Brazil | 19 |
| **11** | Hospital Central da Aeronáutica, Rio de Janeiro, Rio de Janeiro, Brazil | 16 |
| **12** | Hospital Estadual de Doenças Tropicais Dr. Anuar Auad, Anápolis, Goiás, Brazil | 13 |
| **13** | Hospital Geral de Fortaleza, Fortaleza, Ceará, Brazil | 10 |
| **14** | Hospital Mater Dei, Belo Horizonte, Minas Gerais, Brazil | 8 |
| **15** | Santa Casa de Misericórdia de Belo Horizonte, Belo Horizonte, Minas Gerais, Brazil | 6 |
| **16** | Complexo Hospitalar Municipal de São Caetano do Sul, São Caetano do Sul, São Paulo, Brazil | 5 |
| **17** | Complexo do Trabalhador de Curitiba, Curitiba, Paraná, Brazil | 5 |
| **18** | Hospital da Força Aérea de São Paulo, São Paulo, São Paulo, Brazil | 3 |
| **19** | Hospital das Clínicas da Universidade Federal do Pernambuco, Recife, Pernambuco, Brazil | 1 |

**Supplementary Results**

**SMTable 2. Baseline characteristics of the ITT COVID-19 population**

|  | **All patients (*N*=498)** | **Nitazoxanide (*n*=249)** | **Placebo (*n*=249)** | ***P* values between groups*** |
| --- | --- | --- | --- | --- |
| **Age (years), median (IQR)** | 56 (46–67) | 56 (46–68) | 56 (46–66) | .721 |
| **Age range, *n* (%)** |  |  |  | .682 |
| 18–40 years | 72 (14) | 36 (15) | 36 (15) |  |
| 41–59 years | 229 (46) | 110 (44) | 119 (47) |  |
| ≥60 years | 197 (40) | 103 (41) | 94 (38) |  |
| **Male sex, *n* (%)** | 305 (61) | 145 (58) | 160 (64) | .168 |
| **Ethnicity, *n* (%)** |  |  |  | .437 |
| Mixed | 235 (47) | 120 (48) | 115 (46) |  |
| White | 209 (42) | 103 (41) | 106 (43) |  |
| Black | 39 (8) | 18 (7) | 21 (8) |  |
| Asian | 10 (2) | 7 (3) | 3 (1) |  |
| Other | 5 (1) | 1 (1) | 4 (2) |  |
| **BMI, *n* (%)** |  |  |  | .573 |
| <29.9 kg/m^2^ | 324 (65) | 165 (66) | 159 (64) |  |
| ≥30.0 kg/m^2^ | 174 (35) | 84 (34) | 90 (36) |  |
| **Time from symptom onset to randomization (days), median (IQR)** | 7 (5–10) | 8 (5–10) | 7 (5–10) | .274 |
| **Coexisting condition, *n* (%)** |  |  |  |  |
| Hypertension | 178 (36) | 91 (37) | 87 (35) | .708 |
| Diabetes mellitus | 112 (23) | 54 (23) | 58 (23) | .668 |
| Asthma | 9 (2) | 5 (2) | 4 (2) | .737 |
| Chronic obstructive pulmonary disease | 7 (1) | 3 (1) | 4 (2) | .703 |
| Human immunodeficiency virus infection | 3 (1) | 2 (1) | 1 (0.5) | .563 |
| None | 280 (56) | 140 (56) | 140 (56) | .536 |
| **Medications, *n* (%)** |  |  |  |  |
| Angiotensin-II receptor antagonists | 76 (15) | 43 (17) | 33 (13) | .213 |
| Angiotensin-conversing enzyme inhibitors | 93 (19) | 50 (20) | 43 (17) | .421 |
| **Symptom at diagnosis, *n* (%)** |  |  |  |  |
| Dry cough | 405 (81) | 202 (81) | 203 (81) | .908 |
| Productive cough | 45 (9) | 27 (11) | 18 (7) | .160 |
| Sore throat | 9 (2) | 5 (2) | 4 (2) | .737 |
| Shortness of breath | 334 (67) | 175 (70) | 159 (64) | .127 |
| **Clinical parameters at time of admission** |  |  |  |  |
| Temperature (°C), median (IQR) | 36 (36–37) | 36 (36–37) | 36 (36–37) | .952 |
| Respiratory rate (breaths/min), median (IQR) | 20.5 (19–24) | 20 (19–24) | 21 (19–24) | .746 |
| SpO_2_ (%), median (IQR) | 92 (91–93) | 92 (90–93) | 92 (91–93) | .905 |
| **Diagnosis of SARS-CoV-2 infection, *n* (%)** |  |  |  |  |
| Nasopharyngeal swab, positive | 412 (83) | 204 (82) | 208 (84) | .514 |
| **Laboratory parameters at the time of admission** |  |  |  |  |
| Hb (g/dL), median (IQR) | 13.3 (11.9–14.5) | 13.3 (11.8–14.5) | 13.4 (12–14.5) | .623 |
| WBC (×10^3^/mL), median (IQR) | 7.7 (5.9 – 9.8) | 7.9 (6.2–9.9) | 7.5 (5.7–9.8) | .150 |
| Neutrophils (×10^3^/mL), median (IQR) | 5.8 (4.1–7.6) | 5.9 (4.3–7.6) | 5.5 (3.9–7.3) | .141 |
| Lymphocytes (×10^3^/mL), median (IQR) | 1.9 (1.4–2.5) | 1.9 (1.4–2.5) | 1.9 (1.4–2.5) | .515 |
| Platelets (×10^3^/mL), median (IQR) | 218 (1675–286) | 220 (169–288) | 215 (164–279) | .491 |
| C-reactive protein (mg/L), median (IQR) | 113.6 (73–146) | 112.5 (73–145.4) | 115 (74–147) | .538 |
| D-dimer (mg/L), median (IQR) | 1016 (537–1667) | 1054 (564–1667) | 975 (522.5–1676.5) | .384 |
| Ferritin (mg/L), median (IQR) | 443.5 (245–699) | 462 (231–725) | 437 (250–698) | .745 |
| Lactate dehydrogenase (IU/L), median (IQR) | 285 (181–425) | 303 (185–433) | 268 (175–404.5) | .093 |
| **Chest CT score, *n* (%)** |  |  |  | .773 |
| 0%–25% | 0 (0) | 0 (0) | 0 (0) |  |
| 26%–50% | 336 (68) | 165 (66) | 171 (69) |  |
| 51%–75% | 99 (20) | 50 (20) | 49 (20) |  |
| 76%–100% | 63 (13) | 34 (14) | 29 (12) |  |

Coexisting conditions were coded according to the terms used in the Medical Dictionary for Regulatory Activities, version 23.0. Descriptive statistics with simple and relative frequencies for qualitative variables and median and interquartile range for continuous variables. Baseline, immediately before the administration of nitazoxanide or placebo on day 1. Ethnicity was self-reported by the patients.

BMI, body mass index (weight in kilograms divided by the square of the height in meters); COVID-19, coronavirus disease 2019; CT, computed tomography; Hb, hemoglobin; IQR, interquartile range; ITT, intention-to-treat; IU, international unit; SARS-CoV-2, severe acute respiratory syndrome coronavirus 2; WBC, white blood cell count.

**P* value of Pearson’s chi-squared test for qualitative variables or Student’s t test for parametric continuous variables or Mann-Whitney U test for non-parametric continuous variables comparing nitazoxanide with placebo on day 1 (baseline).

**SMTable 3. Baseline characteristics of the mITT-positive COVID-19 population**

|  | **All patients (*N*=367)** | **Nitazoxanide (*N*=183)** | **Placebo (*N*=184)** | ***P* value*** |
| --- | --- | --- | --- | --- |
| **Age (years), median (IQR)** | 56 (47-67) | 56 (47-69) | 56 (45-66) | .381 |
| **Age range, *n.* (%)** |  |  |  | .858 |
| 18 - 40 years | 51 (14) | 24 (13) | 27 (15) |  |
| 41 - 59 years | 168 (46) | 83 (45) | 85 (46) |  |
| ≥60 years | 148 (40) | 76 (42) | 72 (39) |  |
| **Male sex, *n.* (%)** | 231 (63) | 110 (60) | 121 (66) | .262 |
| **Ethnicity, *n.* (%)** |  |  |  | .648 |
| Mixed | 165 (44) | 85 (46) | 80 (44) |  |
| White | 161 (44) | 76 (42) | 85 (45) |  |
| Black | 32 (9) | 16 (9) | 16 (9) |  |
| Asian | 9 (3) | 6 (3) | 3 (2) |  |
| Other | 0 (0) | 0 (0) | 0 (0) |  |
| **BMI, *n.* (%)** |  |  |  | .713 |
| <29.9 kg/m^2^ | 242 (66) | 119 (65) | 123 (67) |  |
| ≥ 30.0 kg/m^2^ | 125 (34) | 64 (35) | 61 (33) |  |
| **Time from symptom onset to randomization (days), median (IQR)** | 7 (5-9) | 7 (5-9) | 6 (5-9) | .278 |
| **Coexisting condition, *n.* (%)** |  |  |  |  |
| Hypertension | 135 (37) | 71 (39) | 64 (35) | .425 |
| Diabetes mellitus | 81 (22) | 40 (22) | 41 (22) | .922 |
| Asthma | 6 (2) | 4 (2) | 2 (1) | .407 |
| Chronic obstructive pulmonary disease | 5 (1) | 3 (2) | 2 (1) | .648 |
| Human immunodeficiency virus infection | 3 (1) | 2 (1) | 1 (0.5) | .559 |
| None | 205 (56) | 100 (55) | 105 (57) | .641 |
| **Concomitant medications, *n.* (%)** |  |  |  |  |
| Angiotensin-II receptor antagonists | 58 (16) | 34 (19) | 24 (13) | .146 |
| Angiotensin-converting enzyme inhibitors | 74 (20) | 41 (22) | 33 (18) | .286 |
| **Symptom at diagnosis, *n.* (%)** |  |  |  |  |
| Dry cough | 300 (82) | 147 (80) | 153 (83) | .484 |
| Productive cough | 31 (8) | 20 (11) | 11 (6) | .088 |
| Sore throat | 4 (1) | 2 (1) | 2 (1) | .996 |
| Shortness of breath | 286 (78) | 153 (84) | 133 (72) | .009 |
| **Clinical parameters at hospital admission** |  |  |  |  |
| Temperature (°C), median (IQR) | 36 (36-37) | 36 (36-37) | 36 (36-37) | .275 |
| Respiratory rate (breaths per min), median (IQR) | 21 (19-24) | 21 (19-25) | 21 (19-24) | .925 |
| SpO_2_ (%) _,_ median (IQR) | 92 (90-93) | 92 (89-93) | 92 (90-93) | .547 |
| **Laboratory parameters at the time of admission** |  |  |  |  |
| Hb (g/dL), median (IQR) | 13.5 (12.1-14.6) | 13.5 (11.8-14.5) | 13.4 (12.2-14.7) | .535 |
| WBC (× 10^3^/mL), median (IQR) | 7.7 (5.9 – 9.8) | 7.8 (6.2 – 9.6) | 7.5 (5.7 – 9.8) | .433 |
| Neutrophils (× 10^3^/mL), median (IQR) | 5.8 (4.2-7.5) | 5.9 (4.3-7.5) | 5.5 (4.0-7.5) | .339 |
| Lymphocytes (× 10^3^/mL), median (IQR) | 1.9 (1.4-2.5) | 1.9 (1.4-2.5) | 1.8 (1.3-2.6) | .766 |
| Platelets (× 10^3^/mL), median (IQR) | 215 (166-284) | 216 (168-283.0) | 215 (165-286) | .998 |
| C-reactive protein (mg/L), median (IQR) | 120 (76-149) | 118 (74-148) | 122 (85-150) | .209 |
| D-dimer (mg/L), median (IQR) | 7 (5-9) | 7 (5-9) | 6 (5-9) | .592 |
| Ferritin (mg/L), median (IQR) | 509 (282-747) | 502 (254-735) | 511 (292-792) | .350 |
| Lactate dehydrogenase (UI/L), median (IQR) | 296 (184-425) | 304 (185-433) | 284 (182-408) | .519 |
| **Chest CT score, no. (%)** |  |  |  | .600 |
| 0% –25% | 0 (0) | 0 (0) | 0 (0) |  |
| 26%–50% | 223 (61) | 109 (60) | 114 (62) |  |
| 51%–75% | 83 (22) | 40 (23) | 43 (23) |  |
| 76%–100% | 61 (17) | 34 (17) | 27 (15) |  |

Coexisting conditions were coded according to the terms used in the Medical Dictionary for Regulatory Activities, version 23.0. Descriptive statistics with simple and relative frequencies for qualitative variables and median and interquartile range for continuous variables. Baseline, immediately before the administration of nitazoxanide or placebo on day 1. Ethnicity was self-reported by the patients.

BMI, body mass index (weight in kilograms divided by the square of the height in meters); COVID-19, coronavirus disease 2019; CT, computed tomography; Hb, hemoglobin; IQR, interquartile range; ITT, intention-to-treat; IU, international unit; SARS-CoV-2, severe acute respiratory syndrome coronavirus 2; WBC, white blood cell count.

**P* value of Pearson’s chi-squared test for qualitative variables or Student’s t test for parametric continuous variables or Mann-Whitney U test for non-parametric continuous variables comparing nitazoxanide with placebo on day 1 (baseline).

**SMTable 4. Comparison of baseline characteristics between the ITT, mITT, and mITT-positive COVID-19 populations**

|  | ***P* value of ITT versus mITT versus mITT-positive*** |
| --- | --- |
| **Age (years)** | .933 |
| **Age range (years)r** | .999 |
| **Sex** | .852 |
| **Ethnicity** | .813 |
| **BMI (kg/m^2^)** | .961 |
| **Time from symptom onset to randomization** | .001 |
| **Coexisting condition** |  |
| Hypertension | .950 |
| Diabetes mellitus | .989 |
| Asthma | .939 |
| Chronic obstructive pulmonary disease | .974 |
| Human immunodeficiency virus infection | .929 |
| None | .990 |
| **Concomitant medications** |  |
| Angiotensin-II receptor antagonists | .858 |
| Angiotensin-converting enzyme inhibitors | .976 |
| **Symptom at diagnosis** |  |
| Dry cough | .968 |
| Productive cough | .930 |
| Sore throat | .691 |
| Shortness of breath | .001 |
| **Clinical parameters at hospital admission** |  |
| Temperature (°C) | .944 |
| Respiratory rate (breaths/min) | .411 |
| SpO_2_ (%) | .415 |
| **Laboratory parameters at hospital admission** |  |
| Hb (g/dL) | .866 |
| WBC (×10^3^/mL) | .932 |
| Neutrophils (×10^3^/mL) | .900 |
| Lymphocytes (×10^3^/mL) | .842 |
| Platelets (×10^3^/mL) | .939 |
| C-reactive protein (mg/L) | .390 |
| D-dimer (mg/L) | .138 |
| Ferritin (mg/L) | .192 |
| Lactate dehydrogenase (IU/L) | .764 |
| Chest CT score | .210 |

Coexisting conditions were coded according to the terms used in the Medical Dictionary for Regulatory Activities, version 23.0. Baseline, immediately before the administration of nitazoxanide or placebo on day 1. Ethnicity was reported by the patients.

BMI, body mass index (weight in kilograms divided by the square of the height in meters); COVID-19, coronavirus disease 2019; CT, computed tomography; Hb, hemoglobin; IU, international unit; WBC, white blood cell count.

**P* value for comparison between three groups according to the type of variable. For qualitative variables, a proportion test with Yates’s correction (more than two categories) was used. For continuous variables, one-way ANOVA (parametric data) or Kruskal-Wallis (non-parametric data) was used to compare these three groups.

**SMTable 5. Comparison of the primary outcome between the mITT, ITT, and mITT-positive populations**

| **Primary outcome ICU admission, *n* (%)** | **mITT (*n*=405)** | | | **ITT (*n*=498)** | | | **mITT-positive (*n*=367)** | | | ***P* value total population**** |
| --- | --- | --- | --- | --- | --- | --- | --- | --- | --- | --- |
|  | **Nitazoxanide (*n*=202)** | **Placebo (*n*=203)** | ***P* value*** | **Nitazoxanide (*n*=249)** | **Placebo (*n*=249)** | ***P* value*** | **Nitazoxanide (*n*=183)** | **Placebo (*n*=184)** | ***P* value*** |  |
| Day 1 | 2 (1) | 4 (2) | .414 | 2 (1) | 4 (2) | .411 | 2 (1) | 4 (2) | .414 | .862 |
| Day 3 | 16 (8) | 18 (9) | .709 | 16 (7) | 18 (8) | .752 | 13 (7) | 13 (7) | .709 | .782 |
| Day 5 | 19 (10) | 21 (11) | .714 | 19 (9) | 21 (10) | .740 | 16 (9) | 16 (9) | .714 | .860 |
| Day 7 | 19 (10) | 27 (14) | .203 | 19 (9) | 27 (13) | .202 | 16 (9) | 22 (12) | .184 | .905 |
| Day 14 | 20 (10) | 29 (15) | .182 | 20 (9) | 29 (15) | .296 | 17 (10) | 24 (13) | .305 | .912 |

ICU, intensive care unit; ITT, intention-to-treat; mITT, modified intention-to-treat.

**P* value of ICU admission day by day comparing nitazoxanide with placebo groups using Pearson’s chi-squared test.

***P* value of ICU admission day by day comparing different populations (mITT, ITT, and mITT-positive) using Fisher’s exact test (adjusted for three factors).

**SMTable 6. Eight-point ordinal scale of clinical status measured daily in the mITT population treated with nitazoxanide or placebo until day 14**

| **Day** | **Eight-point ordinal scale of clinical status** | **Nitazoxanide, *n* (%)** | **Placebo, n (%)** | **% difference** | ***P* value*** |
| --- | --- | --- | --- | --- | --- |
| 1 | 1 and 2: Not hospitalized with no limitations of activities and with limitation of activities, respectively | 0 (0) | 0 (0) | 0.0 | .733 |
|  | 3: Hospitalized, not requiring supplemental oxygen or ongoing medical care | 0 (0) | 0 (0) | 0.0 |  |
|  | 4: Hospitalized, not receiving supplemental oxygen but requiring ongoing medical care | 2 (1) | 2 (1) | 0.0 |  |
|  | 5: Hospitalized, requiring low-flow supplemental oxygen | 170 (84) | 170 (84) | −0.4 |  |
|  | 6: Hospitalized, receiving high-flow oxygen through a nasal cannula | 30 (15) | 29 (14) | −0.6 |  |
|  | 7: Hospitalized, receiving invasive mechanical ventilation | 2 (1) | 2 (1) | 0.0 |  |
|  | 8: Death | 0 (0) | 2 (1) | 1.0 |  |
| 2 | 1 and 2: Not hospitalized with no limitations of activities and with limitation of activities, respectively | 0 (0) | 0 (0) | 0.0 | .005 |
|  | 3: Hospitalized, not requiring supplemental oxygen or ongoing medical care | 0 (0) | 0 (0) | 0.0 |  |
|  | 4: Hospitalized, not receiving supplemental oxygen but requiring ongoing medical care | 42 (21) | 15 (7) | −13.4 |  |
|  | 5: Hospitalized, requiring low-flow supplemental oxygen | 137 (68) | 155 (76) | 8.5 |  |
|  | 6: Hospitalized, receiving high-flow oxygen through a nasal cannula | 13 (6) | 20 (10) | 3.4 |  |
|  | 7: Hospitalized, receiving invasive mechanical ventilation | 9 (5) | 10 (5) | 0.5 |  |
|  | 8: Death | 1 (0.5) | 3 (2) | 1.0 |  |
| 3 | 1 and 2: Not hospitalized with no limitations of activities and with limitation of activities, respectively | 0 (0) | 0 (0) | 0.0 | <.001 |
|  | 3: Hospitalized, not requiring supplemental oxygen or ongoing medical care | 66 (33) | 4 (2) | −30.7 |  |
|  | 4: Hospitalized, not receiving supplemental oxygen but requiring ongoing medical care | 52 (26) | 34 (17) | −9.0 |  |
|  | 5: Hospitalized, requiring low-flow supplemental oxygen | 61 (30) | 132 (65) | 34.8 |  |
|  | 6: Hospitalized, receiving high-flow oxygen through a nasal cannula | 6 (3) | 12 (6) | 2.9 |  |
|  | 7: Hospitalized, receiving invasive mechanical ventilation | 16 (8) | 18 (9) | 0.9 |  |
|  | 8: Death | 1 (0.5) | 3 (2) | 1.0 |  |
| 4 | 1 and 2: Not hospitalized with no limitations of activities and with limitation of activities, respectively | 62 (31) | 23 (11) | −19.4 | <.001 |
|  | 3: Hospitalized, not requiring supplemental oxygen or ongoing medical care | 63 (31) | 4 (2) | −29.2 |  |
|  | 4: Hospitalized, not receiving supplemental oxygen but requiring ongoing medical care | 44 (22) | 46 (23) | 0.9 |  |
|  | 5: Hospitalized, requiring low-flow supplemental oxygen | 11 (5) | 98 (48) | 42.9 |  |
|  | 6: Hospitalized, receiving high-flow oxygen through a nasal cannula | 2 (1) | 8 (4) | 2.9 |  |
|  | 7: Hospitalized, receiving invasive mechanical ventilation | 19 (9) | 20 (10) | 0.5 |  |
|  | 8: Death | 1 (0.5) | 4 (2) | 1.5 |  |
| 5 | 1 and 2: Not hospitalized with no limitations of activities and with limitation of activities, respectively | 124 (61) | 65 (32) | −29.4 | <.001 |
|  | 3: Hospitalized, not requiring supplemental oxygen or ongoing medical care | 37 (18) | 22 (11) | −7.5 |  |
|  | 4: Hospitalized, not receiving supplemental oxygen but requiring ongoing medical care | 15 (7) | 22 (11) | 3.4 |  |
|  | 5: Hospitalized, requiring low-flow supplemental oxygen | 4 (2) | 64 (32) | 29.5 |  |
|  | 6: Hospitalized, receiving high-flow oxygen through a nasal cannula | 2 (1) | 5 (3) | 1.5 |  |
|  | 7: Hospitalized, receiving invasive mechanical ventilation | 19 (9) | 21 (10) | 0.9 |  |
|  | 8: Death | 1 (0.5) | 4 (2) | 1.5 |  |
| 6 | 1 and 2: Not hospitalized with no limitations of activities and with limitation of activities, respectively | 140 (69) | 82 (40) | −28.9 | <.001 |
|  | 3: Hospitalized, not requiring supplemental oxygen or ongoing medical care | 32 (16) | 24 (12) | −4.0 |  |
|  | 4: Hospitalized, not receiving supplemental oxygen but requiring ongoing medical care | 6 (3) | 38 (19) | 15.7 |  |
|  | 5: Hospitalized, requiring low-flow supplemental oxygen | 1 (0.5) | 28 (14) | 13.3 |  |
|  | 6: Hospitalized, receiving high-flow oxygen through a nasal cannula | 1 (0.5) | 4 (2) | 1.5 |  |
|  | 7: Hospitalized, receiving invasive mechanical ventilation | 19 (9) | 23 (11) | 1.9 |  |
|  | 8: Death | 3 (2) | 4 (2) | 0.5 |  |
| 7 | 1 and 2: Not hospitalized with no limitations of activities and with limitation of activities, respectively | 144 (71) | 131 (65) | −6.8 | .028 |
|  | 3: Hospitalized, not requiring supplemental oxygen or ongoing medical care | 31 (15) | 20 (10) | −5.4 |  |
|  | 4: Hospitalized, not receiving supplemental oxygen but requiring ongoing medical care | 3 (2) | 12 (6) | 4.4 |  |
|  | 5: Hospitalized, requiring low-flow supplemental oxygen | 1 (0.5) | 6 (3) | 2.5 |  |
|  | 6: Hospitalized, receiving high-flow oxygen through a nasal cannula | 1 (0.5) | 2 (1) | 0.5 |  |
|  | 7: Hospitalized, receiving invasive mechanical ventilation | 19 (9) | 27 (13) | 3.9 |  |
|  | 8: Death | 3 (2) | 5 (3) | 1.0 |  |
| 14 | 1 and 2: Not hospitalized with no limitations of activities and with limitation of activities, respectively | 163 (81) | 162 (80) | −0.9 | .121 |
|  | 3: Hospitalized, not requiring supplemental oxygen or ongoing medical care | 13 (6) | 5 (3) | −3.9 |  |
|  | 4: Hospitalized, not receiving supplemental oxygen but requiring ongoing medical care | 0 (0) | 2 (1) | 1.0 |  |
|  | 5: Hospitalized, requiring low-flow supplemental oxygen | 0 (0) | 0 (0) | 0.0 |  |
|  | 6: Hospitalized, receiving high-flow oxygen through a nasal cannula | 0 (0) | 0 (0) | 0.0 |  |
|  | 7: Hospitalized, receiving invasive mechanical ventilation | 20 (10) | 29 (14) | 4.4 |  |
|  | 8: Death | 6 (3) | 5 (3) | −0.5 |  |

mITT, modified intention-to-treat.

**P* value comparing all status day by day between nitazoxanide and placebo.

**SMTable 7. Supplemental oxygen therapy requirements in the mITT population**

| **Day** | **Oxygen requirement** | **Nitazoxanide, *n* (%)** | **Placebo, *n* (%)** | **% difference** | ***P* value*** |
| --- | --- | --- | --- | --- | --- |
| 1 | Not receiving oxygen | 0 (0) | 0 (0) | 0.0 | – |
|  | 1 L O_2_/min | 0 (0) | 0 (0) | 0.0 | – |
|  | 1–5 L O_2_/min | 171 (85) | 170 (84) | 0.9 | .802 |
|  | High-flow oxygen through a nasal cannula | 29 (14) | 29 (14) | 0.1 | .904 |
|  | Invasive mechanical ventilation | 2 (1) | 4 (2) | −1.0 | .414 |
| 2 | Not receiving oxygen | 0 (0) | 0 (0) | 0.0 | – |
|  | 1 L O_2_/min | 42 (21) | 15 (7) | 13.4 | <.001 |
|  | 1–5 L O_2_/min | 137 (68) | 155 (76) | −8.5 | .056 |
|  | High-flow oxygen through a nasal cannula | 13 (6) | 20 (10) | −3.4 | .209 |
|  | Invasive mechanical ventilation | 10 (5) | 13 (6) | −1.5 | .527 |
| 3 | Not receiving oxygen | 66 (33) | 4 (2) | 30.7 | <.001 |
|  | 1 L O_2_/min | 52 (26) | 34 (17) | 9.0 | .030 |
|  | 1–5 L O_2_/min | 61 (30) | 132 (65) | −34.8 | <.001 |
|  | High-flow oxygen through a nasal cannula | 6 (3) | 12 (6) | −2.9 | .145 |
|  | Invasive mechanical ventilation | 16 (8) | 18 (9) | −0.9 | .709 |
| 4 | Not receiving oxygen | 125 (62) | 27 (13) | 48.6 | <.001 |
|  | 1 L O_2_/min | 44 (22) | 46 (23) | −0.9 | .790 |
|  | 1–5 L O_2_/min | 11 (5) | 98 (48) | −42.8 | <.001 |
|  | High-flow oxygen through nasal cannula | 2 (1) | 8 (4) | −3.0 | .054 |
|  | Invasive mechanical ventilation | 19 (9) | 21 (10) | −0.9 | .726 |
| 5 | Not receiving oxygen | 161 (80) | 87 (43) | 36.8 | <.001 |
|  | 1 L O_2_/min | 15 (7) | 22 (11) | −3.4 | .215 |
|  | 1–5 L O_2_/min | 4 (2) | 64 (32) | −29.5 | <.001 |
|  | High-flow oxygen through a nasal cannula | 2 (1) | 5 (2) | −1.5 | .247 |
|  | Invasive mechanical ventilation | 19 (9) | 21 (10) | −0.9 | .714 |
| 6 | Not receiving oxygen | 173 (86) | 117 (58) | 28.0 | <.001 |
|  | 1 L O_2_/min | 5 (2) | 27 (13) | −10.8 | <.001 |
|  | 1–5 L O_2_/min | 1 (0.5) | 27 (13) | −12.8 | <.001 |
|  | High-flow oxygen through a nasal cannula | 1 (0.5) | 3 (2) | −1.0 | .310 |
|  | Invasive mechanical ventilation | 21 (10) | 25 (12) | −2.4 | .388 |
| 7 | Not receiving oxygen | 175 (87) | 151 (74) | 12.2 | .002 |
|  | 1 L O_2_/min | 3 (2) | 12 (6) | −4.4 | .018 |
|  | 1–5 L O_2_/min | 1 (0.5) | 6 (3) | −2.5 | .057 |
|  | High-flow oxygen through a nasal cannula | 1 (0.5) | 2 (1) | −0.5 | .562 |
|  | Invasive mechanical ventilation | 19 (9) | 27 (13) | −3.9 | .203 |
| 14 | Not receiving oxygen | 176 (90) | 165 (84) | 5.8 | .110 |
|  | 1 L O_2_/min | 0 (0) | 4 (2) | −2.0 | .044 |
|  | 1–5 L O_2_/min | 0 (0) | 0 (0) | 0.0 | - |
|  | High-flow oxygen through nasal cannula | 0 (0) | 0 (0) | 0.0 | - |
|  | Invasive mechanical ventilation | 20 (10) | 29 (14) | −4.4 | .182 |

Eleven patients died and are thus not included in the table.

mITT, modified intention-to-treat.

**P* value comparing nitazoxanide with placebo according to the use or non-use of each type of ventilatory oxygen at each day.

**SMTable 8. Symptoms in the nitazoxanide and placebo groups at different time points**

| **Day** |  | **Nitazoxanide (*n*=202), *n* (%)** | **Placebo (*n*=203), *n* (%)** | ***P* value between groups*** |
| --- | --- | --- | --- | --- |
| 1 | All symptoms | 202 (100) | 203 (100) | 1.000 |
|  | Dry cough | 163 (81) | 169 (83) | .503 |
|  | Productive cough | 21 (10) | 13 (6) | .147 |
|  | Sore throat | 3 (2) | 3 (2) | .995 |
|  | Shortness of breath | 162 (80) | 142 (70) | .017 |
| 2 | All symptoms | 200 (99) | 200 (98.5) | .657 |
|  | Dry cough | 163 (81) | 169 (83) | .659 |
|  | Productive cough | 16 (8) | 13 (6) | .766 |
|  | Sore throat | 5 (2) | 5 (2) | .906 |
|  | Shortness of breath | 100 (50) | 137 (68) | .001 |
| 3 | All symptoms | 191 (95) | 190 (94) | .683 |
|  | Dry cough | 164 (81) | 157 (77) | .632 |
|  | Productive cough | 9 (4) | 10 (5) | .892 |
|  | Sore throat | 7 (4) | 3 (2) | .409 |
|  | Shortness of breath | 62 (31) | 128 (63) | <.001 |
| 4 | All symptoms | 64 (32) | 155 (76) | <.001 |
|  | Dry cough | 55 (27) | 117 (58) | <.001 |
|  | Productive cough | 4 (2) | 11 (5) | <.001 |
|  | Sore throat | 1 (0.5) | 6 (3) | <.001 |
|  | Shortness of breath | 14 (7) | 71 (35) | <.001 |
| 5 | All symptoms | 24 (12) | 103 (51) | <.001 |
|  | Dry cough | 21 (10) | 82 (40) | <.001 |
|  | Productive cough | 2 (1) | 9 (4) | <.001 |
|  | Sore throat | 1 (0.5) | 5 (2) | <.001 |
|  | Shortness of breath | 3 (2) | 33 (16) | <.001 |
| 6 | All symptoms | 20 (10) | 78 (38) | <.001 |
|  | Dry cough | 18 (9) | 63 (31) | <.001 |
|  | Productive cough | 1 (0.5) | 5 (2) | <.001 |
|  | Sore throat | 1 (0.5) | 4 (2) | <.001 |
|  | Shortness of breath | 2 (1) | 21 (10) | <.001 |
| 7 | All symptoms | 25 (12) | 133 (66) | <.001 |
|  | Dry cough | 23 (11) | 125 (62) | <.001 |
|  | Productive cough | 3 (2) | 7 (3) | .275 |
|  | Sore throat | 0 (0) | 7 (3) | .017 |
|  | Shortness of breath | 4 (2) | 10 (5) | .159 |
| 14 | All symptoms | 10 (5) | 95 (47) | <.001 |
|  | Dry cough | 9 (4) | 95 (47) | <.001 |
|  | Productive cough | 0 (0) | 0 (0) | .162 |
|  | Sore throat | 0 (0) | 0 (0) | .162 |
|  | Shortness of breath | 1 (0.5) | 0 (0) | .234 |

**P* value based on chi-squared test comparing the presence and absence of each symptom between the nitazoxanide and placebo groups at each day.

**SMTable 9. Medications received during the study in the mITT population**

|  | **All data (*n*=405), *n* (%)** | **Nitazoxanide (*n*=202), *n* (%)** | **Placebo (*n*=203), *n* (%)** | ***P* value between groups** |
| --- | --- | --- | --- | --- |
| **Antibiotic** | 66 (16) | 33 (16) | 33 (16) | .983 |
| **Anticoagulant** |  |  |  |  |
| Day 1 | 266 (66) | 169 (65) | 134 (66) | .888 |
| Day 3 | 315 (83) | 163 (85) | 152 (80) | .168 |
| Day 7 | 60 (17) | 25 (14) | 35 (20) | .119 |
| **Corticosteroids** |  |  |  |  |
| Day 1 | 219 (54) | 108 (54) | 111 (55) | .765 |
| Day 3 | 178 (47) | 90 (47) | 88 (47) | .913 |
| Day 7 | 98 (28) | 48 (26) | 50 (29) | .665 |

mITT, modified intention-to-treat.

**P* value comparing use and non-use of each medication between groups on each day. Chi-squared test was used to compare the use of medications during hospitalization in each group.

**SMTable 10. Viral load and RT-PCR status in the ITT, mITT, and mITT-positive populations**

|  | **Nitazoxanide** | | **Placebo** | | ***P* value between days: nitazoxanide*** | ***P* value between days: placebo*** | ***P* value between groups: day 1**** | ***P* value between groups: day 7**** |
| --- | --- | --- | --- | --- | --- | --- | --- | --- |
|  | **Day 1** | **Day 7** | **Day 1** | **Day 7** |  |  |  |  |
| **ITT population (*n*=498)** | | | | | | | | |
| Positive RT-PCR status, *n* (%) | 204 (82) | 69 (28) | 208 (84) | 75 (30) | <.001 | .001 | .565 | .322 |
| **mITT population (*n*=405)** | | | | | | | | |
| Positive RT-PCR status, *n* (%) | 183 (91) | 68 (34) | 184 (91) | 75 (37) | .001 | .001 | .987 | .311 |
| **mITT-positive population (n=367)** | | | | | | | | |
| Nasopharyngeal swab RT-PCR viral load (log_10_copies/mL), median (IQR) |  |  |  |  |  |  |  |  |
| All patients | 4.9 (3.4-6.3) | 0.1 (0.1-3.5) | 4.9 (3.6-6.3) | 0.1 (0.1-4.2) | <.001 | <.001 | .890 | .083 |
| 0–5 days† | 5.2 (3.8-6.5) | 0.1 (0.1-3.1) | 4.9 (3.5-6.2) | 0.1 (0.1-3.9) | <.001 | <.001 | .318 | .210 |
| 6–10 days† | 4.7 (3.3-6.2) | 0.1 (0.1-3.5) | 4.9 (3.8-6.0) | 0.1 (0.1-4.2) | <.001 | <.001 | .652 | .502 |
| >10 days † | 5.4 (3.4-5.7) | 0.1 (0.1-4.1) | 5.3 (3.3-6.9) | 2.7 (0.1-5.1) | <.001 | <.001 | .890 | .083 |
| Positive RT-PCR status, *n* (%) | – | 68 (37) | – | 75 (41) | – | – | – | .330 |
| Change in viral load day 1 to day 7, median (IQR) | −96 (−98 to −32) | | −94 (−98 to −17.6) | | – | – | – | .136 |

IQR, interquartile range; ITT, intention-to-treat; mITT, modified intention-to-treat; RT-PCR, reverse-transcriptase polymerase chain reaction. **P* value based on McNemar test to evaluate the change in each group and population.

***P* value based on chi-squared test to evaluate differences in proportions between groups in each population.

†Time from symptom onset to randomization in days.

**SMTable 11.** **Nasopharyngeal swab RT-PCR viral load stratified according to its level on day 1 (baseline)**

| **mITT-positive (*n*=367)** | | **Nitazoxanide** | | **Placebo** | | ***P* value between groups at day 7** | |
| --- | --- | --- | --- | --- | --- | --- | --- |
|  |  | **Day 7** | | **Day 7** | |  |  |
| Nasopharyngeal swab RT-PCR viral load (log_10_ copies/mL), median (IQR) |  | |  | |  | |  |
| Day 1: until 3.45 | 0.1 (0.1–0.1) | | 0.1 (0.1–1.9) | | .528 | |  |
| Day 1: 3.46 to 4.91 | 0.1 (0.1–3.2) | | 0.1 (0.1–3.3) | | .874 | |  |
| Day 1: 4.92 to 6.28 | 0.1 (0.1–3.5) | | 2.5 (0.1–4.6) | | .299 | |  |
| Day 1: >6.29 | 3.3 (0.1–5.2) | | 4.8 (2.7–6.3) | | .054 | |  |

Note that a higher viral load at baseline was associated with a tendency toward greater reduction in nitazoxanide compared with placebo.

IQR, interquartile range; mITT-positive, modified intention-to-treat positive; RT-PCR, reverse transcriptase polymerase chain reaction.

**P* value based on Mann-Whitney U test to compare viral load on day 7 between groups at each level of viral load on day 1 (baseline).

**SMTable 12. Secondary outcomes in the mITT population**

|  | **Nitazoxanide (*n*=202)** | | | **Placebo (*n*=203)** | | | ***P* value between time points: nitazoxanide*** | ***P* value between time points: placebo*** | ***P* value between groups at day 1*** | ***P* value between groups at day 3*** | ***P* value between groups at day 7*** |
| --- | --- | --- | --- | --- | --- | --- | --- | --- | --- | --- | --- |
|  | **Day 1** | **Day 3** | **Day 7** | **Day 1** | **Day 3** | **Day 7** |  |  |  |  |  |
| **Clinical parameters** | | | | | | | | | | | |
| Temperature (°C), median (IQR) | 36 (36–37) | 36 (36–36) | 36 (36–37) | 36 (36–37) | 36 (36–37) | 36 (36–37) | .085 | .092 | .419 | .145 | .810 |
| Respiratory rate (breaths/min), median (IQR) | 21 (19–24) | 19 (18–21) | 19 (17–20) | 21 (19–24) | 19 (18–21) | 18 (17–20) | **<.001** | **<.001** | .819 | .260 | .632 |
| SpO_2_ (%), median (IQR) | 92 (89–93) | 93 (92–94) | 95 (94–95) | 92 (90–93) | 93 (92–94) | 94 (94–95) | **<.001** | **<.001** | .588 | **.012** | .108 |
| **Laboratory parameters** | | | | | | | | | | | |
| Hb (g/dL), median (IQR) | 14 (12–15) | 14 (12–15) | 14 (12–15) | 13 (12–15) | 13 (12–14) | 14 (12–15) | .699 | **.012** | .613 | .322 | .583 |
| WBC (×10^3^/mL), median (IQR) | 7.9 (6.3–9.8) | 7.8 (6.0–9.8) | 8.0 (6.5–10.1) | 7.5 (5.8–9.8) | 8.1 (6.1–10.8) | 8.6 (6.6–10.6) | .085 | **.001** | .199 | .489 | .414 |
| Neutrophils (×10^3^/mL), median (IQR) | 6 (4–8) | 6 (4–8) | 6 (4–8) | 5 (4–7) | 6 (4–8) | 6 (4–8) | .474 | .468 | .154 | .824 | .948 |
| Lymphocytes (×10^3^/mL), median (IQR) | 2 (1–2) | 2 (1–3) | 2 (2–3) | 2 (1–3) | 2 (2–3) | 2 (2–3) | **<.001** | **<.001** | .570 | .116 | .174 |
| Platelets (×10^3^/mL), median (IQR) | 218 (169–286) | 247 (192–312) | 282 (219–319) | 216 (165–290) | 246 (195–314) | 268 (200–311) | **<.001** | **<.001** | .934 | .751 | .149 |
| C-reactive protein (mg/L), median (IQR) | 116 (74–146) | 67 (42–91) | 22 (15–34) | 121 (85–149) | 93 (66–124) | 38 (22–63) | **<.001** | **<.001** | .225 | **<.001** | **<.001** |
| D-dimer (mg/L), median (IQR) | 1152 (592–1686) | 826 (503–1239) | 525 (311–897) | 1121 (545–1956) | 863 (512–1620) | 805 (425–1467) | **<.001** | **<.001** | .829 | .101 | **<.001** |
| Ferritin (mg/L), median (IQR) | 485 (240–747) | 406 (210–596) | 290 (174–524) | 480 (273–727) | 456 (252–698) | 402 (234–622) | **<.001** | **<.001** | .822 | .070 | **.002** |
| Lactate dehydrogenase (IU/L), median (IQR) | 300 (184–433) | 300 (181–429) | 202 (124–286) | 270 (180–397) | 269 (174–397) | 232 (160–345) | **<.001** | **.009** | .269 | .316 | **.001** |

Statistical analysis was performed using modified two-way ANOVA for non-parametric data, considering group and time effects at the same time. Interactions between group–time were tested and *p* values are presented when interactions were observed.

Hb, hemoglobin; IQR, interquartile range; ITT, intention-to-treat; IU, international unit; SpO_2_, oxygen saturation; WBC, white blood cell count.

**SMTable 13. Abnormal findings on chest CT scans in the mITT population**

|  | **Chest CT** | **Nitazoxanide (*n*=202), *n* (%)** | **Placebo (*n*=203), *n* (%)** | ***P* values between groups*** |
| --- | --- | --- | --- | --- |
| Day 1 | 0%–25% | 0 (0) | 0 (0) | .764 |
|  | 26%–50% | 121 (60) | 127 (63) |  |
|  | 51%–75% | 47 (23) | 47 (23) |  |
|  | 76%–100% | 34 (17) | 29 (14) |  |
| Day 3 | 0%–25% | 11 (7) | 4 (2) | .019 |
|  | 26%–50% | 106 (68) | 100 (60) |  |
|  | 51%–75% | 27 (17) | 35 (21) |  |
|  | 76%–100% | 12 (8) | 27 (16) |  |
| Day 7 | 0%–25% | 63 (37) | 20 (12) | <.001 |
|  | 26%–50% | 103 (61) | 105 (65) |  |
|  | 51%–75% | 2 (1) | 32 (20) |  |
|  | 76%–100% | 0 (0) | 4 (3) |  |
| *P* value between days** | | 0.042 | 0.313 |  |

On day 3, 83 patients (46 in the nitazoxanide group and 37 in the placebo group) and on day 7, 76 patients (34 in the nitazoxanide group and 42 in the placebo group) did not have a chest CT scan.

CT, computed tomography; mITT, modified intention-to-treat.

**P* value comparing proportions of patients between groups in each category of chest CT day by day.

***P* value comparing proportions of patients between days in the same group in each category of chest CT.

**SMTable 14. Adverse events in the ITT population**

|  | **Nitazoxanide (*n*=249), *n* (%)** | **Placebo (*n*=249), *n* (%)** | ***P* value*** |
| --- | --- | --- | --- |
| Number of participants with at least one adverse event | 79 (32) | 79 (32) | 1.000 |
| Number of participants with two adverse events | 22 (9) | 25 (10) | .646 |
| Number of participants with three or more adverse events | 27 (11) | 23 (9) | .551 |
| **Detailed adverse events** |  |  |  |
| Diarrhea | 23 (9) | 18 (7) | .415 |
| Headache | 38 (15) | 53 (21) | .082 |
| Nausea | 20 (8) | 15 (6) | .381 |
| Abdominal pain | 12 (5) | 15 (6) | .553 |
| Abnormal color of urine | 8 (3) | 6 (2) | .588 |
| Vomiting | 3 (1) | 0 (0) | .082 |
| Pruritus | 3 (1) | 2 (1) | .653 |
| Urticaria | 4 (2) | 4 (2) | 1.000 |

All adverse events reported after nitazoxanide or placebo.

ITT, intention-to-treat.

**P* value comparing proportions of adverse events between nitazoxanide and placebo.

**SMTable 15. Comparison of primary outcome occurrence between selected characteristics, independent of treatment**

|  | ICU admission (IMV) | | | | OR (95% CI) | *P* value |
| --- | --- | --- | --- | --- | --- | --- |
|  | Yes | | No | |  |  |
|  | *n* | Row % | *N* | Row % |  |  |
| **Age range** |  |  |  |  |  |  |
| ≤60 years | 23 | 14.4 | 137 | 85.6 | 1.36 (0.75–2.46) | .317 |
| >60 years | 27 | 11.0 | 218 | 89.0 | * |  |
| **BMI** |  |  |  |  |  |  |
| <30 kg/m^2^ | 30 | 11.4 | 234 | 88.6 | 0.78 (0.42–1.42) | .412 |
| ≥30 kg/m^2^ | 20 | 14.2 | 121 | 85.8 | * |  |
| **Sex** |  |  |  |  |  |  |
| Female | 19 | 12.1 | 138 | 87.9 | 0.96 (0.52–1.77) | .906 |
| Male | 31 | 12.5 | 217 | 87.5 | * |  |
| **Time from symptom onset until hospital admission** |  |  |  |  |  |  |
| ≤7 days | 29 | 12.1 | 210 | 87.9 | 0.95 (0.52–1.74) | .876 |
| >7 days | 21 | 12.7 | 145 | 87.3 | * |  |
| **SpO_2_ at hospital admission** |  |  |  |  |  |  |
| ≤90% | 36 | 32.7 | 74 | 67.3 | 9.76 (5.00–19.05) | <.001 |
| >90% | 14 | 4.7 | 281 | 95.3 | * |  |
| **Chest CT scan at hospital admission** |  |  |  |  |  |  |
| 26%–50% | 5 | 2.0 | 243 | 98.0 | * |  |
| 51%–75% | 21 | 22.3 | 73 | 77.7 | 13.98 (5.09–38.38) | <.001 |
| 76%–100% | 24 | 38.1 | 39 | 61.9 | 29.91 (10.77–83.03) | <.001 |
| **Corticosteroids during hospitalization** |  |  |  |  |  |  |
| Yes | 36 | 14.9 | 206 | 85.1 | 1.86 (0.97– 3.57) | .062 |
| No | 14 | 8.6 | 149 | 91.4 | * |  |

*P* value was calculated using chi-squared test with Yates correction when necessary.

BMI, body mass index; CI, confidence interval; CT, computerized tomography; ICU, intensive care unit; IMV, invasive mechanical ventilation; OR, odds ratio; SpO_2_, peripheral saturation of oxygen.

*Reference category for OR calculation.


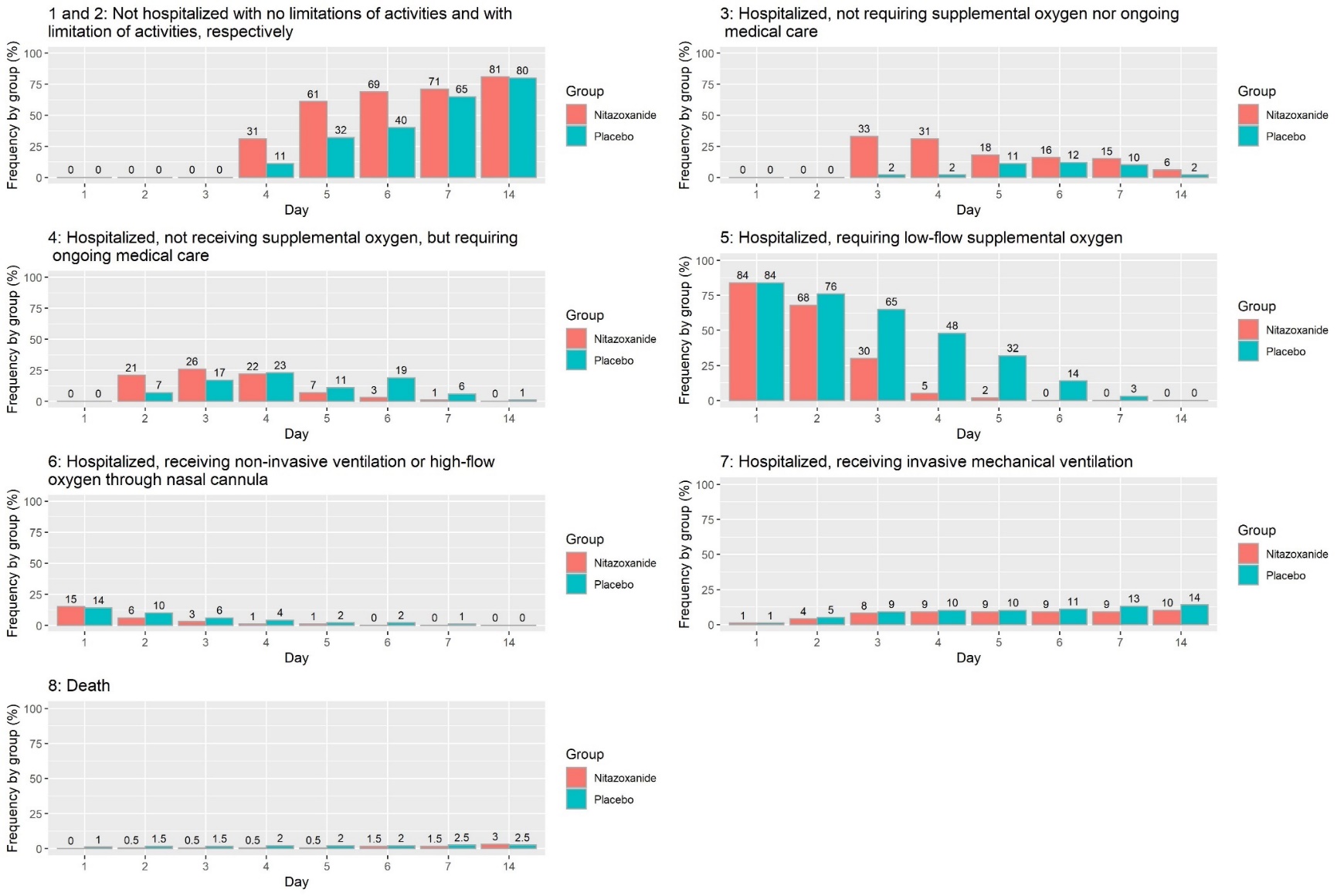


**SMFigure 1. Categories on an eight-point ordinal scale in the nitazoxanide and placebo groups at different time points.** Frequency of patients on an eight-point ordinal scale of clinical status in the mITT population treated with nitazoxanide or placebo until day 14. The numbers above the bars are the percentage of patients.


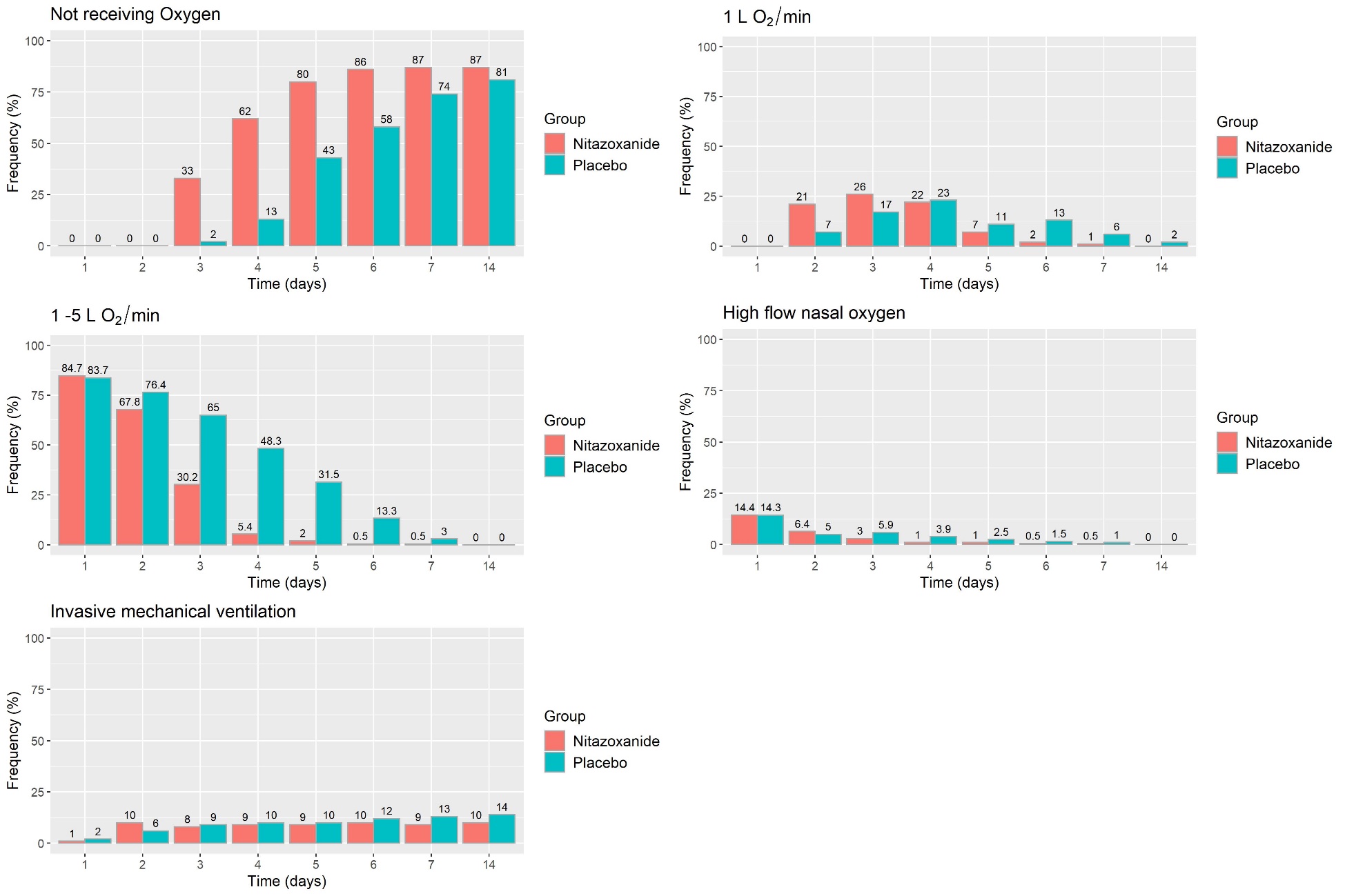


**SMFigure 2. Oxygen requirement in the nitazoxanide and placebo groups at different time points.** Frequency of patients not receiving oxygen, receiving 1 L and 1–5 L of oxygen/min, and high-flow oxygen through a nasal cannula.

and invasive mechanical ventilation in mITT population treated with nitazoxanide or placebo until day 14. Each number above the bars represents the percentage of patients.


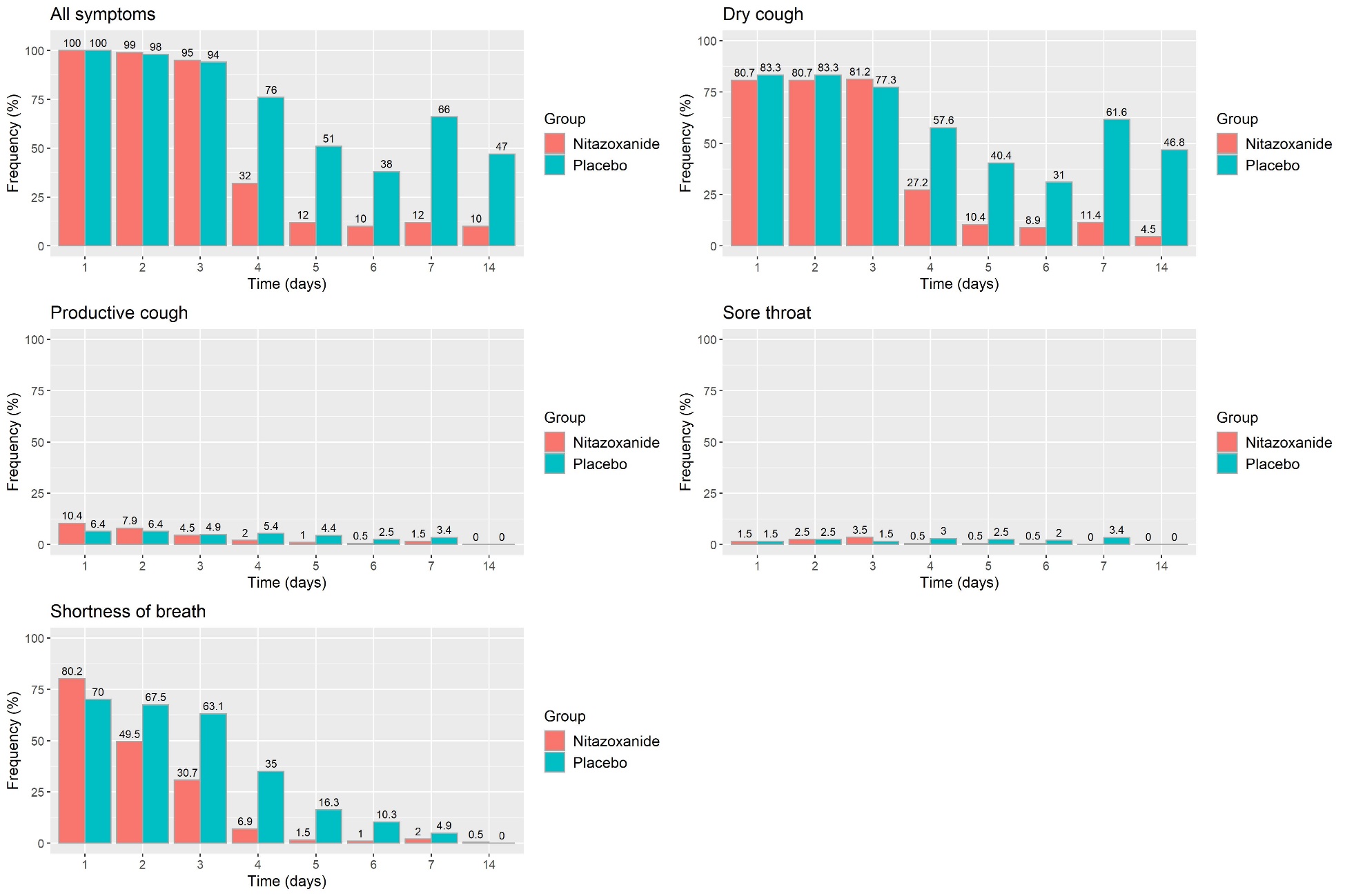


**SMFigure 3. COVID-19 symptoms in the nitazoxanide and placebo groups at different time points.** Frequency of patients presenting at least one symptom, dry cough, productive cough, sore throat, and shortness of breath in the mITT population treated with nitazoxanide or placebo until day 14. The numbers above the bars are the percentage of patients. mITT, modified intention-to-treat.

**References**

Franquet T. Imaging of pulmonary viral pneumonia. Radiology 2011;260(1):18–39.

Hansell DM, Bankier AA, MacMahon H, McLoud TC, Müller NL, Remy J. Fleischner Society: glossary of terms for thoracic imaging. Radiology 2008;246(3):697–722.

Kalil AC, Patterson TF, Mehta AK, Tomashek KM, Wolfe CR, Ghazaryan MD, et al. Baricitinib plus remdesivir for hospitalized adults with Covid-19. N Engl J Med 2021;384:795-807.

Rocco PRM, Silva PL, Cruz FF, et al. Early use of nitazoxanide in mild Covid-19 disease: randomised, placebo-controlled trial. Eur Respir J 2021;58:2003725.
